# Ultrasound-assisted deep eutectic solvent extraction of tyrosinase inhibitors from lotus seed peel powder

**DOI:** 10.1016/j.ultsonch.2026.107944

**Published:** 2026-06-30

**Authors:** Chengheng Zhang, Jin Liu, Jingjing Tan, Jiangtao Cai, Ying Long, Senwen Deng, Shiyin Guo, Changwei Liu

**Affiliations:** a Hunan Engineering Research Center of Lotus Deep Processing and Nutritional Health Sciences, Hunan Key Laboratory of Economic Crops Genetic Improvement and Integrated Utilization, School of Life and Health Sciences, Hunan University of Science and Technology, Xiangtan 411201, China; b School of Resource & Environment and Safety Engineering, Hunan University of Science and Technology, Xiangtan 411201, China; c Yuelushan Laboratory of Hunan Province, Changsha 410004, China

**Keywords:** Ultrasound-assisted extraction, Deep eutectic solvent, Lotus seed peel powder, Tyrosinase Inhibitor

## Abstract

Lotus seed peel powder (LSP), a major by-product of lotus seed processing, is rich in bioactive constituents but remains largely underutilized. Conventional aqueous and ethanolic extraction methods for LSP are constrained by low extraction efficiency and poor selectivity. In this study, an ultrasound-assisted deep eutectic solvent (DES) extraction (UADE) strategy was developed to recover tyrosinase inhibitors from LSP. UADE significantly enhanced flavonoid and polyphenol extraction compared with traditional methods. Among the tested DESs, the L-proline/lactic acid system exhibited the highest selectivity for tyrosinase inhibitors. Using response surface methodology, the optimal extraction conditions were determined as follows: Pro to LA 1:2, 20 % water, solid-to-liquid ratio 1:40 (w/v), 47 °C, 300 W, 70 min. Under these conditions, the tyrosinase inhibition rate reached 96.51 %, and the half-maximal inhibitory concentration (IC_50_) of the purified extract was 1.02 mg/mL. In addition, the DES system retained good reusability over multiple extraction cycles. Mechanistic analyses revealed that ultrasonic treatment markedly disrupted LSP cell wall structure, promoting the release of active components. Four key tyrosinase inhibitors, including isorhamnetin-3-O-galactoside-6′’-rhamnoside, were identified via UHPLC-QE-Orbitrap-MS analysis combined with molecular docking and molecular dynamics simulations. DFT calculations suggested that hydrogen bonding and π-π stacking interactions between DES components and key inhibitors were the core driving forces of extraction selectivity. Zebrafish assays confirmed that the extract inhibited tyrosinase activity *in vivo* and downregulated the expression of multiple melanogenesis-related genes. These findings establish UADE as an effective and selective approach for the extraction of tyrosinase inhibitors from natural resources, providing a methodological basis for developing anti-melanogenic cosmetic ingredients.

## Graphical abstract

**Figure:**
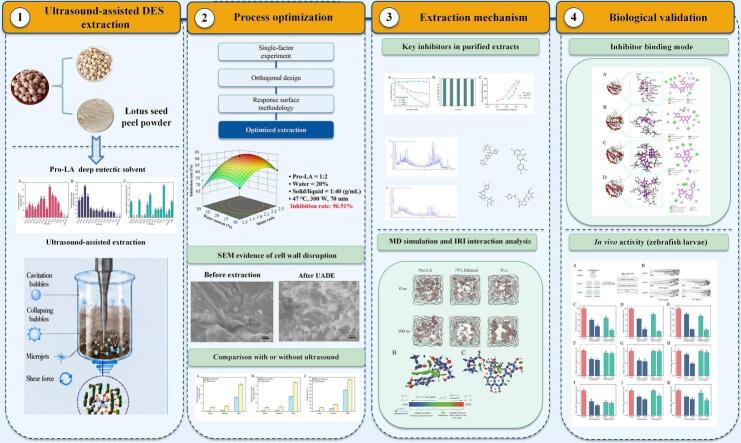

## 1 Introduction

Lotus seed (*Nelumbo nucifera* Gaertn.) is widely recognized as both a functional food and herbal medicine in Asia [1]. The mature lotus seeds have a red seed coat that imparts a notable bitterness, necessitating its mechanical removal during industrial processing. This procedure generates a major by-product, known as lotus seed peel powder (LSP), which typically constitutes 15–20 % of the total seed mass [2]. LSP is abundant in bioactive components, including polyphenols, polysaccharides, and proteins, and exhibits antioxidant, anti-inflammatory, and hypoglycemic properties [3], [4], [5]. Despite its beneficial attributes, LSP is primarily utilized as a low-grade animal feed and remains largely underexploited in mainstream applications [6].

Tyrosinase (TYR) is a key rate-limiting enzyme in the melanin biosynthesis pathway, found in mammals and various other organisms [7]. Research indicates that abnormal tyrosinase activity is significantly associated with multiple pigmentary disorders, including freckles, melasma, and solar lentigines [8]. Consequently, the development of effective tyrosinase inhibitors has emerged as a therapeutic strategy for treating pigmentary skin disorders. Currently, tyrosinase inhibitors can be classified into two main categories based on their origin: natural bioactive compounds and synthetic chemicals [9], [10]. Natural plant-derived products, in particular, have garnered considerable attention due to their superior biocompatibility and low toxicity when compared to synthetic inhibitors [11], [12], [13], [14].

The extraction of bioactive compounds from agricultural by-products such as LSP primarily relies on conventional methods including hot water extraction, Soxhlet extraction, and organic solvent reflux extraction [15]. However, these conventional approaches are inherently limited by low extraction efficiency, harsh operating conditions (e.g., high temperatures), high costs, and environmental concerns [16]. A less frequently recognized limitation is that conventional solvents lack inherent molecular selectivity, leading to the non-specific co-extraction of large quantities of impurities, which not only substantially increases downstream purification costs but also compromises the purity and quality of the final product [17]. These limitations have significantly hindered the industrial-scale production of high-quality natural tyrosinase inhibitors from agricultural by-products.

Ultrasound-assisted extraction (UAE) is an energy-efficient technology that enhances bioactive yield and quality by enabling faster processing with reduced solvent and energy consumption [18]. Ultrasonic cavitation generates extreme local temperatures and pressures and exerts multiple physical mechanisms (fragmentation, erosion, sonoporation, shear forces and detexturation) that disrupt plant cellular structures, enhance mass transfer, and significantly improve bioactive compound extraction yields [17], [18]. The efficacy of UAE, however, relies not only on the solvent's ability to dissolve target compounds, but also on its physical properties, which critically influence the cavitation dynamics and thereby the overall extraction efficiency [19]. Deep eutectic solvents (DESs), which are homogeneous mixtures formed by hydrogen bond donors (HBDs) and hydrogen bond acceptors (HBAs), present a promising alternative. DESs offer advantages over traditional solvents, including low volatility, low toxicity, and biodegradability. By tailoring the HBD/HBA combinations, DESs can facilitate the selective extraction of target components [17]. DES properties synergize with ultrasound-induced cavitation to enhance mass transfer and increase the extraction efficiency of bioactive compounds (anthocyanins, flavonoids, polyphenols) from plant materials, offering a greener and more efficient alternative to conventional methods [20], [21], [22]. Ultrasound-assisted DES extraction (UADE) has been applied to extract tyrosinase inhibitors [23], [24], [25]; however, two critical limitations remain in the current literature. First, research has predominantly focused on maximizing extraction yield, whereas extraction selectivity, a critical parameter governing downstream purification costs and final product purity, has received far less systematic attention. Second, mechanistic investigations are largely limited to macroscopic, phenomenological observations. Specifically, the molecular-level interactions, particularly the specific bonding mechanisms between the complex hydrogen-bond networks of DES and the active inhibitor molecules, are still poorly understood.

This study presents the first successful development of an efficient and selective UADE method for extracting tyrosinase inhibitors from LSP. This innovative approach overcomes the key limitations of conventional extraction techniques, including low efficiency and poor selectivity. The resulting extract underwent comprehensive phytochemical characterization to validate its composition, along with *in vivo* bioactivity assessment to confirm its functional efficacy. These findings have significant potential to enhance the valorization of LSP and broaden the applicability of UADE in the food, cosmetic, and pharmaceutical industries.

## 2 Materials and methods

### 2.1 Chemicals and reagents

Lotus seed peel powder was provided by Liangyuan Food Co., Ltd. (Xiangtan, China). Tyrosinase (mushroom) and arbutin were purchased from Coolaber Technology Co., Ltd. (Beijing, China). Rutin trihydrate and gallic acid were purchased from Aladdin Biochemical Technology Co., Ltd. (Shanghai, China). Choline chloride (99 %)**,** betaine (98 %), L-proline (99 %), glucose (99 %), lactic acid (88 %), xylitol (99 %), malic acid (98 %), urea (99 %), citric acid (99.5 %), glycerol (99 %), 1,4-butanediol (99 %), ethylene glycol (99.5 %), macroporous adsorption resins (AB-8, D101, YPR-Ⅱ, NKA-9), and L-3,4-dihydroxyphenylalanine (L-DOPA) were supplied by Macklin Biochemical Technology Co., Ltd. (Shanghai, China). Real-time quantitative PCR kits were provided by Vazyme Biotech Co., Ltd. (Nanjing, China). Wild-type AB strain zebrafish were supplied by Shanghai FishBio Co., Ltd. (Shanghai, China). Primers were synthesized by Sangon Biotech Co., Ltd. (Shanghai, China). All other reagents were of analytical grade.

### 2.2 Ultrasound-assisted DES extraction

#### 2.2.1 Screening of DESs

The DESs used in this study were selected and prepared as previously described [26]. Thirteen DESs with specific compositions were prepared (Table S1). Sieved LSP (100 mesh) was mixed with DESs at a solid-to-liquid ratio of 1:20 (w/v), and subjected to ultrasound-assisted extraction at 300 W, 40 kHz and 40 °C for 90 min (SN-16D-40, Guangdong Skymen Ultrasonic Industrial Co., Ltd., Shenzhen, Guangdong, China). After centrifugation at 10,000 × g for 10 min (Heraeus Multifuge X1R, Thermo Fisher Scientific, Waltham, MA, USA), the supernatant was collected for further analysis.

#### 2.2.2 Determination of total phenolic and flavonoid content

The total phenolic content (TPC) was determined using the Folin-Ciocalteu method [27] as described in a previous study. 125 μL of Folin–Ciocalteu reagent was mixed with 50 μL of LSP extract. Subsequently, 1.25 mL of 7 % (w/v) Na_2_CO_3_ solution was added, and the volume was adjusted to 3 mL with ultrapure water, followed by incubation at room temperature for 90 min. The optical density of each sample was measured by a UV–Vis spectrophotometer (Cary 60, Agilent Technologies, Inc., Santa Clara, CA, USA) at 760 nm. The TPC was expressed as milligrams of gallic acid equivalents (GAE) per gram of dry weight (mg GAE/g DW). The total flavonoid content (TFC) was quantified by the aluminum chloride colorimetry method according to a previously described method with minor modifications [28]. Briefly, 10 μL of the LSP extract was diluted to 1 mL with 70 % methanol solution. Then, 0.1 mL of the diluted extract was mixed with 40 µL of 0.5 M NaNO_2_ solution, and the mixture was kept at room temperature for 5 min. Subsequently, 40 µL of 0.3 M AlCl_3_ solution was added, and the mixture was incubated for a further 6 min. Thereafter, 400 µL of 0.5 M NaOH solution was added, and the total volume was adjusted to 1 mL using 70 % methanol solution. The solution was incubated at 35 °C for 30 min in the dark. The absorbance was measured at 510 nm by the same UV–Vis spectrophotometer The TFC was expressed as milligrams of rutin equivalents (RE) per gram of dry weight (mg RE/g DW).

#### 2.2.3 Determination of tyrosinase inhibition rate

Tyrosinase activity was assayed using a previously described method with minor modifications [29]. The reaction mixture comprised 100 µL of 100 mM phosphate buffer (pH 7.0), 80 µL of 20 mM L-DOPA, 2 µL of inhibitor, and 10 µL of mushroom tyrosinase (1000 U/mL). After incubation at 37 ℃ for 20 min, the reaction was terminated by adding 400 µL of 0.2 M Na_2_CO_3_ solution. Absorbance was measured at 475 nm using an Agilent Cary 60 UV–Vis spectrophotometer (Agilent Technologies, Santa Clara, CA, USA). One unit of tyrosinase activity was defined as the amount of enzyme required to catalyze the formation of 1 nmol dopachrome per minute. The inhibition rate was calculated relative to the blank group (reaction mixture without inhibitor), with arbutin (0.6 mg/mL) as the positive control. The tyrosinase inhibition rate was calculated using the following formula:

$$Inhibition\left(\%\right)=\left[\left(A_{c}-A_{s}\right)/A_{c}\right]\times 100,$$

where A_s_ and A_c_ represent the absorbance of the sample and the negative control, respectively.

#### 2.2.4 Optimization of extraction conditions

To determine the optimal extraction conditions, single-factor experiments were performed initially. The variables and their corresponding tested levels were as follows: the molar ratio of L-proline to lactic acid (1:1, 1:1.5, 1:2, 1:2.5, 1:3), water content (10 %, 20 %, 30 %, 40 %, 50 %, 60 %, v/v), solid-to-liquid ratio (1:10, 1:20, 1:30, 1:40, 1:50, w/v), extraction temperature (20 °C, 30 °C, 40 °C, 50 °C, 60 °C, 70 ℃), ultrasonic power (50, 100, 150, 200, 250, 300 W), and extraction time (50, 60, 70, 80, 90, 100, 110 min). The optimal level for each factor was selected based on the highest tyrosinase inhibition rate.

A six-factor, four-level orthogonal design was established to identify significant factors, facilitating a more focused and efficient response surface methodology (RSM) optimization of the key variables (Table S2). The results indicated that ultrasonic power was the least statistically significant factor and had the weakest influence on the tyrosinase inhibition rate under the tested conditions (Tables S3-S4). The highest average inhibition rate was obtained when ultrasonic power was at level 4, corresponding to 300 W. Therefore, 300 W was selected as the fixed ultrasonic power for the subsequent optimization. For the solid-to-liquid ratio, the highest average inhibition rate was obtained at level 4, corresponding to 1:40 (w/v). Thus, considering its non-significant effect and optimal average response at this level, the solid-to-liquid ratio was fixed at 1:40 (w/v) for subsequent optimization. Following this, a four-factor, three-level RSM analysis was conducted, which further revealed that extraction time was not statistically significant (*P* > 0.05) (Tables S5-S7). Accordingly, the extraction time was set at 70 min. Finally, a three-factor, three-level RSM was conducted to optimize the extraction conditions (Tables S8-S10).

### 2.3 Recovery of DESs and purification of inhibitors

10 mL of the extract was diluted to 50 mL with ultrapure water, and mixed with 20 g pre-treated resin in a triangular vial. The mixture was incubated at 25 °C with shaking at 150 rpm. Samples were collected at 0, 3, 6, 9, 12, 15, 18, 21, and 24 h. After centrifugation at 10,000 × g for 10 min (Heraeus Multifuge X1R, Thermo Fisher Scientific, Waltham, MA, USA), the tyrosinase inhibition rate in the supernatant was measured to identify the optimal resin

The recovery of DES and inhibitors was performed following a previously described method [26]. The mixture was transferred to a column, which was initially eluted with 2 bed volumes (BV) of ultrapure water at a flow rate of 1 BV/h to remove unadsorbed components and DES. The fraction eluted by 60 % ethanol solution (v/v) was concentrated via rotary evaporation under reduced pressure, lyophilized, and stored as a powdered extract at −80 ℃. The aqueous eluate containing DESs was then evaporated and dried for DES recovery. The half-maximal inhibitory concentration (IC_50_) was derived from non-linear regression analysis of the inhibition-concentration curves.

### 2.4 Efficacy evaluation of phytochemical enrichment methods

The solvent system that combines ultrasonication with DES was systematically evaluated against conventional solvent-based extraction protocols to assess its efficacy in disrupting plant matrices and releasing bioactive components. For instance, ultrasonic extraction of LSP was conducted under identical conditions using either 75 % (v/v) ethanol or pure water as the extraction medium (solid-to-liquid ratio 1:40 (w/v), 47 °C, 70 min). Morphological alterations in the dehydrated plant matrices were examined using a Motic EasyNanoscan™ scanning electron microscope (SEM, Motic China Group Co., Ltd., Xiamen, China) at a magnification of 5,000 × . Tyrosinase inhibition rate, total flavonoid content (TFC), and total phenolic content (TPC) of the extract were assayed as described in 2.2.2, 2.2.3.

### 2.5 UHPLC-QE-Orbitrap-MS analysis of the extract

Chromatographic separation was performed on a Thermo Scientific UltiMate 3000 UHPLC system (Thermo Fisher Scientific, Waltham, MA, USA) coupled with a Waters HSS T3 column (100 mm × 2.1 mm, 1.8 µm). The column temperature was maintained at 40 ℃, with an injection volume of 5 µL and a flow rate of 0.4 mL/min. The mobile phase comprised 0.1 % formic acid in water (solvent A) and 0.1 % formic acid in acetonitrile (solvent B). A gradient elution program was employed as follows [30]: 0–1.5 min, 95 % A; 1.5–2.5 min, 95 % to 90 % A; 2.5–14 min, 90 % to 60 % A; 14–22 min, 60 % to 5 % A; 22–23 min, 5 % A; 23–23.1 min, 5 % to 95 % A; 23.1–30 min, 95 % A.

Mass spectrometric analysis was performed using an AB Sciex X500R Triple TOF™ mass spectrometer (Sciex, Toronto, Canada). Data acquisition was carried out in both positive and negative electrospray ionization (ESI) modes (Fig. S1). During each acquisition cycle, the ions with intensities greater than 100 were selected for MS/MS analysis. MS^1^ scan: 50–1200 mass-to-charge ratio (*m*/*z*) range, collision energy 30 eV, 10 MS^2^ spectra collected per 50 ms. ESI conditions: Nebulizer gas (GS1) 60 psi, auxiliary gas 60 psi, curtain gas 35 psi, ion source temperature 65 °C. The spray voltage was set to + 5000 V for positive ion mode and − 4000 V for negative ion mode. Raw data were converted to mzML format using ProteoWizard software (version 3.0). Peak identification, filtration, and alignment were executed in MS-DIAL, generating a data matrix containing *m*/*z*, retention time, and intensity values. Compound annotation was performed by matching accurate mass (mass error ≤ 30 ppm) and MS/MS spectra against spectral databases (MassBank, GNPS, RIKEN PlaSMA, BMDMS-NP, and mzCloud), requiring a minimum spectral match score of 80 for confident identification.

### 2.6 Molecular docking and molecular dynamics simulations of potential tyrosinase inhibitors

The crystallographic structure of mushroom tyrosinase (PDB ID: 2Y9X) was obtained from the RCSB Protein Data Bank (https://www.rcsb.org/). The three-dimensional structures of ligand compounds were downloaded from PubChem (https://pubchem.ncbi.nlm.nih.gov/). The top 58 compounds identified in the extract, along with the tyrosinase protein, were prepared using AutoDockTools 1.5.6 (Scripps Research Institute, La Jolla, CA, USA) to generate energy-minimized conformations. Druggable binding pockets on the tyrosinase surface were predicted using the PrankWeb platform (https://prankweb.cz/). Molecular docking was performed with AutoDock Vina, and ligand-receptor binding affinities were evaluated based on docking scores (kcal/mol). The binding poses and intermolecular interactions were visualized and analyzed using Discovery Studio 4.5 Client (BIOVIA, San Diego, CA, USA).

Molecular dynamics (MD) simulations were performed on the optimal docked complex. The ligand structure was converted to PDB format using PyMOL (https://github.com/cgohlke/pymol-open-source-wheels), with hydrogen atoms added and saved in MOL2 format. Topology files were generated via ACPYPE (https://bio2byte.be/acpype/). The simulation system was constructed in GROMACS 2023.2 [31]. A solvated water box model was established, with parameters assigned via the Amber14sb force field. Solvation was implemented using the TIP3P water model, followed by the addition of Na^+^/Cl^−^ ions to neutralize the system charge. Energy minimization was performed to eliminate unfavorable atomic contacts and spatial conflicts. Subsequently, NVT/NPT equilibration was performed for 100 ps each. The V-rescale method was used for temperature coupling, and the C-rescale method was applied for pressure coupling. A 200 ns molecular dynamics simulation was performed at a constant temperature of 300 K, with trajectory frames recorded every 10 ps for subsequent analysis.

### 2.7 Molecular dynamics analysis of dissolution behavior and intermolecular interaction

Initial molecular structures of small molecules were acquired from the PubChem database (https://pubchem.ncbi.nlm.nih.gov/) via their Compound CID identifiers, with 3D structural data downloaded in SDF format. Geometry optimization was performed using Density Functional Theory (DFT) in Gaussian 16, employing the B3LYP functional [32] and the 6-31G(d) basis set under the SMD solvation model. In AmberTools 23.6 [33], the optimized structures were subsequently parameterized using the antechamber module [34] with the GAFF force field [35] where atomic charges were derived via the RESP fitting method [36]. Topology conversion to GROMACS-compatible format was achieved using ACPYPE 2023.10.17 [37], enabling full-atom force field construction for molecular dynamics simulations.

Three distinct systems (DES, 75 % ethanol, and water) were generated using Packmol in cubic boxes of 10 nm × 10 nm × 10 nm [38]. The mixture of identified tyrosinase inhibitors (isorhamnetin-3-O-galactoside-6″-rhamnoside, quercetin, luteolin-8-C-glucoside, and agnuside, 5 molecules for each) was added at first. Molecular dynamics simulations were subsequently performed with the GROMACS 2023.4 package [31]. Energy minimization was conducted via the steepest descent method under periodic boundary conditions, followed by initial NVT ensemble equilibration for 2,000,000 steps (4 ns, time of step: 2 fs) using LINCS hydrogen-bond constraints with temperature maintained at 315.15 K via the V-rescale thermostat. Subsequent NPT ensemble equilibration employed the Berendsen barostat at 1.0 bar for 5,000,000 steps (10 ns, time of step: 2 fs). Production simulations extended for 500,000,000 steps (100 ns) at identical V-rescale-controlled temperature (315.15 K) and pressure (1 bar), with molecular modeling visualized using the VMD package via Tcl scripting (https://www.ks.uiuc.edu/Research/vmd/) [39].

Intermolecular interactions were investigated at the quantum chemistry level through DFT-based theoretical methods in Gaussian 16. The target complex was extracted from the final conformation of molecular dynamics simulations (.gro file), and intermolecular interaction energies were calculated at the M06-2X/def2-TZVP level of theory with BSSE correction using the counterpoise method [40]. Subsequent wavefunction analysis was performed using Multiwfn [41] to visualize weak interaction regions via the Interaction Region Indicator (IRI) method [42].

### 2.8 Whitening efficacy of the extract

#### 2.8.1 Zebrafish experiment

Zebrafish embryos (6–8 h post-fertilization, hpf) were divided into five groups (n = 40 per group; three replicates): the negative control group, the LSP extract treated groups (0.025 and 0.05 mg/mL), and the arbutin treated groups (0.025 and 0.05 mg/mL). Embryos were maintained in petri dishes at 25 °C for 96 h, and medium was replaced every 24 h [43]. All procedures were approved by the Animal Care and Use Committee of Hunan University of Science and Technology (approval number: 20240901).

#### 2.8.2 Determination of body surface pigment content

Zebrafish larvae were exposed to the test compound (0.025 and 0.05 mg/mL extract or arbutin) for 96 h, with minor modifications to the previously described method [43]. After complete hatching, the larvae were lightly anesthetized in 0.1 % (w/v) tricaine methanesulfonate (MS-222) solution and immediately subjected to morphometric analysis using a fluorescence microscope (DMI8, Leica, Wetzlar, Germany). High-resolution images were captured to document melanin distribution and body morphology. Total melanin spot area was quantified using ImageJ software (v1.53) with the following calculation:

$$\text{Re}lativePigmentContent\left(\%\right)=\left(S_{s}/S_{c}\right)\times 100$$

where S_c_ and S_s_ represent the pigment of the negative control group and the sample group, respectively.

#### 2.8.3 Determination of *in vivo* tyrosinase activity

Thirty zebrafish per experimental group were randomly selected for tyrosinase activity analysis [44]. The procedures were conducted as follows: Anesthetized zebrafish were homogenized in ice-cold PBS containing 1 % Triton X-100, and ultrasonicated in an ice-water bath for 5 min. Lysates were centrifuged at 10000 × g for 15 min at 4 °C. The supernatant was collected as a crude tyrosinase extract. Protein concentration was quantified using the BCA method. Tyrosinase activity (U/mg protein) was calculated from the initial linear slope of the reaction curve. The inhibition rate was determined according to Section 2.2.3.

#### 2.8.4 Measurement of melanin content

Melanin content was adapted from Hwang et al. with minor modifications [45]. The centrifuged pellet was resuspended and homogenized in 600 μL of 1 M NaOH solution containing 10 % DMSO, and then incubated at 80 °C for 2 h to achieve complete melanin solubilization. After cooling to room temperature (25 °C), the absorbance of the solution was then measured at 490 nm. The relative melanin content *in vivo* was calculated using the following formula:

$$\text{Re}lativeMelaninContent\left(\%\right)=(A_{s}/A_{c)}\times 100$$

where As and Ac are absorbance of a sample and the negative control, respectively.

#### 2.8.5 Quantitative real-time PCR

After 96-hour treatment, fifty zebrafish were euthanized with 0.1 % (w/v) MS-222 solution, followed by three washes with ice-cold PBS. Total RNA was extracted and reverse-transcribed to cDNA using the BioFast SimplyP Total RNA Extraction Kit (Bioflux Bio, Zhejiang, China). Primer sequences are listed in Table S11 [46]. Genes related to melanin synthesis were specifically analyzed. Reverse transcription was performed using the PrimeScript™ RT Reagent Kit (TaKaRa, Dalian, China), and quantitative PCR (qPCR) was conducted with the TB Green® Premix Ex Taq Kit (TaKaRa, Dalian, China) according to the manufacturer's protocol. Relative gene expressions were normalized to glyceraldehyde-3-phosphate dehydrogenase (*gapdh)* as the internal reference gene, calculated via 2^−ΔΔCT^ method.

### 2.9 Data processing and analysis

All experiments were performed in triplicate. Data are presented as mean ± standard deviation (SD). Data visualization was performed using Origin2018 (OriginLab Corporation, Northampton, MA, USA). Statistical significance analyses were conducted using IBM SPSS Statistics version 17.0 software (IBM SPSS Inc., Armonk, NY, USA). Statistical significance was defined as *P* < 0.05.

## 3 Results

### 3.1 Screening of DESs

**Fig. 1:**
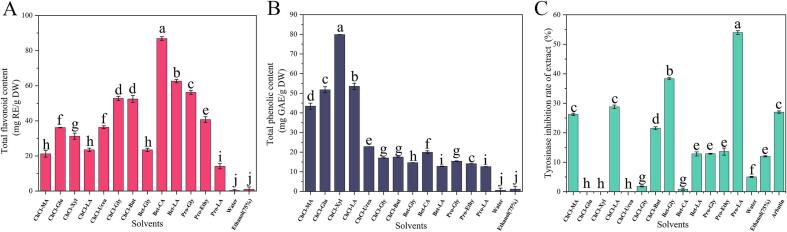
Effect of solvent type on the extraction of bioactive components from LSP. (A) Total flavonoid content of the extracts (TFC, mg RE/g DW), (B) Total phenolic content of the extracts (TPC, mg GAE/g DW), (C) Tyrosinase inhibitory activity of the extracts. Data are presented as mean ± SD (n = 3). Different lowercase letters indicate statistically significant differences (*P* < 0.05). RE, rutin equivalents; DW, dry weight; GAE, gallic acid equivalents.

The selection of solvents is critical for optimizing extraction efficiency. Thirteen DESs reported in our previous research were selected for high extraction efficiencies and selectivity[26]. Compared to conventional solvents such as water and ethanol, DESs demonstrated a significantly enhanced ability to extract flavonoids and polyphenolic compounds from LSP. Aqueous and alcoholic extracts exhibited low TFC and TPC, both measuring below 2 mg/g DW. In contrast, all DES-based extracts exhibited significantly higher levels of both TFC and TPC compared to their aqueous and alcoholic counterparts (*P* < 0.05). Notably, Bet-CA achieved a TFC of 86.87 mg RE/g DW **(**Fig. 1A), while ChCl-Xyl recorded a TPC of 79.88 mg GAE/g DW (Fig. 1B).

The aqueous and alcoholic extracts exhibited tyrosinase inhibition rates of 5.01 % and 12.02 %, respectively, while arbutin (0.5 mg/mL) exhibited 27.78 % inhibition. Notably, the Pro-LA extract demonstrated the highest inhibition rate at 53.98 % (Fig. 1C). Quantitative analysis revealed that the TFC and TPC values of the Pro-LA extract were 14.13 mg RE/g DW (Fig. 1A) and 12.61 mg GAE/g DW, respectively (Fig. 1B), which were the lowest among all tested DESs. Given its superior inhibition activity and high selectivity, the Pro-LA solvent system was selected for subsequent experiments.

### 3.2 Optimization of UADE conditions

**Fig. 2:**
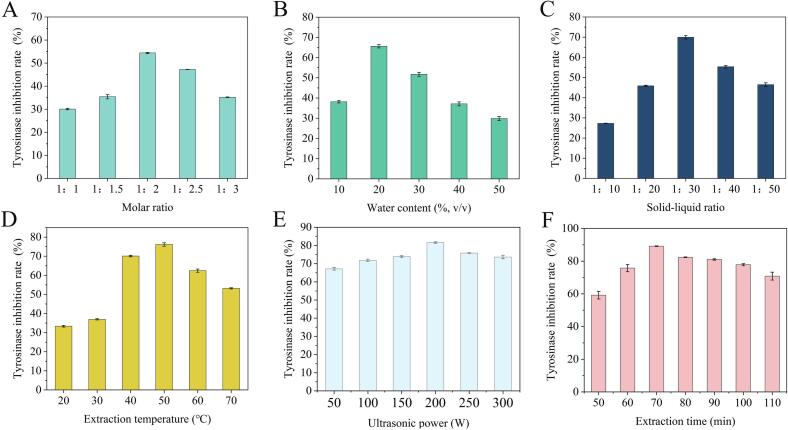
Single-factor optimization for extracting tyrosinase inhibitors from LSP. (A) Effect of the molar ratio of L-proline to lactate, (B) Effect of water content, (C) Effect of the solid-to-liquid ratio, (D) Effect of the extraction temperature, (E) Effect of the ultrasonic power, (F) Effect of the extraction time. Data are expressed as mean ± SD (n = 3).

The molar ratio of L-proline to lactic acid significantly influenced the tyrosinase inhibition rate (Fig. 2A**)**. The inhibition rate increased gradually with the molar ratio, but declined sharply beyond a 1:2 ratio, which was determined to be optimal. In parallel, water content critically affected the activity of extracts: inhibition rates increased proportionally from 10 % to 20 %, but decreased beyond 20 % (Fig. 2B). Therefore, a water content of 20 % was selected as optimal. For the solid-to-liquid ratio, the highest inhibition rate was observed at 1:30 (w/v) (Fig. 2C). Temperature effects indicated that maximal inhibition occurred at 50 °C, while efficiency decreased at temperatures above 50 °C (Fig. 2D). In addition, ultrasonic power affected the extraction efficiency, with the inhibition rate increasing up to 200 W and then decreasing at higher powers **(**Fig. 2E**).** Thus, 200 W was selected as the optimal ultrasonic power. Optimization of ultrasonication time revealed increased inhibition up to 70 min (Fig. 2F), after which prolonged exposure likely led to the degradation of active compounds or an increase in impurity dissolution. Consequently, 70 min was established as the optimal duration. .

**Fig. 3:**
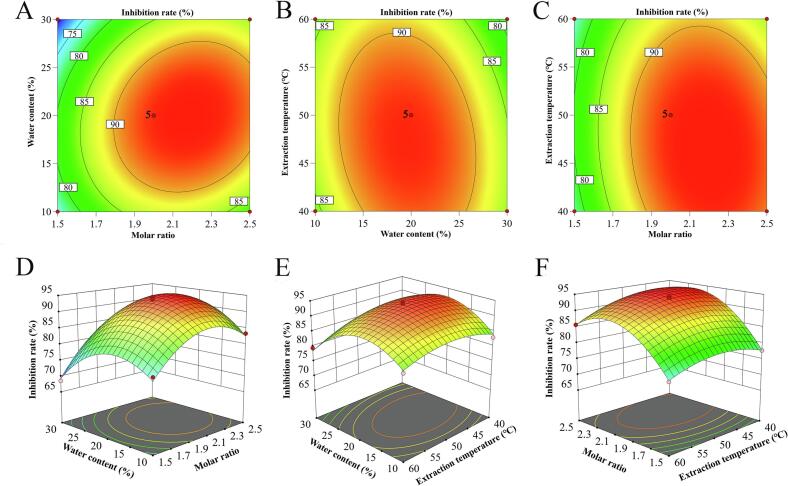
Response surface analysis of the effects of molar ratio, water content, and extraction temperature on the inhibition rate of extracts. (A and D) Interaction between molar ratio and water content. (B and E) Interaction between water content and extraction temperature. (C and F) Interaction between molar ratio and extraction temperature.

A six-factor, four-level orthogonal design was further established to identify the key factors affecting the extraction of tyrosinase inhibitors (Table S2). The range analysis showed that the influence of the six factors followed the order of A > B > F > D > C > E, indicating that the molar ratio of L-proline to lactic acid had the greatest effect on the tyrosinase inhibition rate, whereas ultrasonic power had the weakest effect (Table S3). The ANOVA results (Table S4) further confirmed that only factor A was statistically significant (*P* < 0.0001), while the effects of water content, solid-to-liquid ratio, extraction temperature, ultrasonic power, and extraction time were not significant (*P* > 0.05). Based on the mean inhibition rates at different levels, the optimal combination was determined as A3B2C4D2E4F3, corresponding to an L-proline/lactic acid molar ratio of 1:2, water content of 20 %, solid-to-liquid ratio of 1:40 (w/v), extraction temperature of 50 °C, ultrasonic power of 300 W, and extraction time of 70 min. Among the non-significant factors, ultrasonic power and the solid-to-liquid ratio showed the weakest effects, as indicated by their relatively low F-values and high p-values. Although the single-factor experiment suggested that 200 W produced the highest inhibition rate, the orthogonal test assessed the average effects of the factors under multi-factor combinations. The highest average inhibition rate was obtained when ultrasonic power was at 300 W. Therefore, ultrasonic power was fixed at 300 W for subsequent optimization. For solid-to-liquid ratio, the highest average inhibition rate for this factor was obtained at 1:40. Thus, the solid-to-liquid ratio was ultimately fixed at 1:40. In the first round of RSM testing, which employed a four-factor, three-level design, extraction time was also found to be non-significant (Tables S5-7). The optimization results for TYR inhibitor extraction parameters in the second round of RSM testing are summarized in Table S9. The derived second-order regression model is expressed as follows: Y = 93.54 + 5.64A − 1.03B − 1.71C + 2.34AB − 1.09AC − 1.59BC − 7.66A^2^ − 7.62B^2^ − 3.09C^2^, where A (molar ratio), B (water content), and C (extraction temperature) represent the independent variables. Analysis of variance (ANOVA) confirmed model significance (*P* < 0.0001), with a non-significant lack of fit (*P* = 0.1022) (Table S10**)**. The coefficient of determination (R^2^) and adjusted R^2^ (R^2^_Adj_) were 0.9893 and 0.9755, respectively, with a coefficient of variation (CV) of 1.40 %, which is below the acceptable threshold of 5 %, suggesting superior predictive capability and reproducibility of the model. Based on F-value analysis, the factors influencing extraction were ranked as follows: A (Molar ratio) > C (Extraction temperature) > B (Water content). Both the molar ratio (A) and extraction temperature (C) were identified as dominant factors (*P* < 0.01). A:B and B:C interactions showed significant effects (*P* < 0.05). Three-dimensional response surface plots (Fig. 3) illustrated interactions: A:B (Fig. 3A) and B:C (Fig. 3B) displayed curvature which indicated synergistic effects, whereas A:C interactions exhibited flat profiles (Fig. 3C). The optimal conditions identified through the desirability function were molar ratio of 1:2.18, water content of 20.14 %, and extraction temperature of 47.27 °C. The extraction conditions were slightly modified as follows: molar ratio: 1:2, water content: 20 %, extraction temperature: 47 °C. The experimental inhibition rate was measured at 96.51 ± 0.30 %, which closely aligned with the predicted value of 94.74 %, thereby demonstrating the stability of the model.

### 3.3 Comparison of the UADE method with other extraction methods

**Fig. 4:**
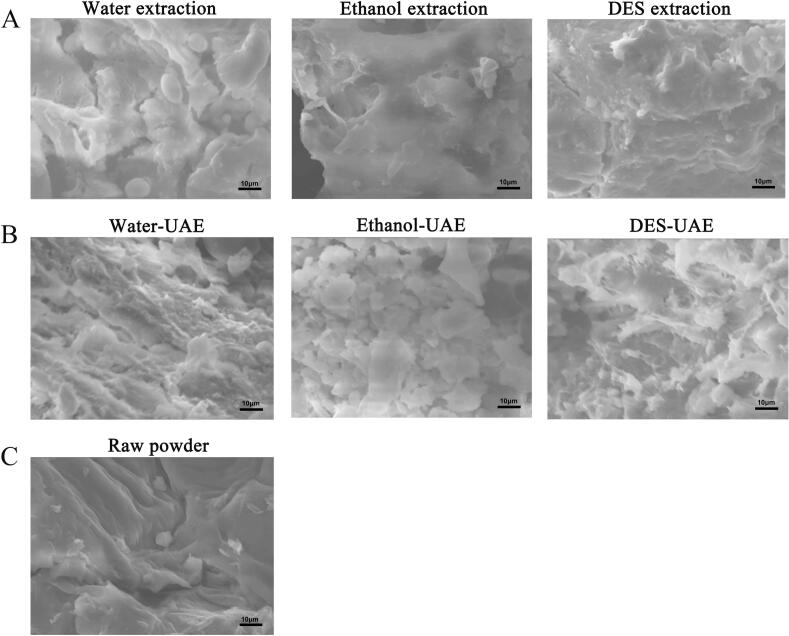
Morphological characterization of LSP with and without ultrasound treatment in different solvents. (A) Non-sonicated group, (B) Ultrasound-assisted extraction (UAE) group. (C) Raw LSP powder. All images were captured at 5000 × magnification; Scale bar: 10 μm.

Fig. 4A-C illustrate the surface morphological characteristics of LSP following various extraction methods. The untreated LSP powder exhibited a smooth and unbroken surface morphology (Fig. 4**C**). In contrast, samples subjected to water, ethanol, or DES extraction, exhibited fractured surfaces, with the most pronounced damage observed in the DES-treated samples (Fig. 4**A**). Water-ultrasonic and ethanol-ultrasonic treatments resulted in more significant surface alterations (Fig. 4**B**). As depicted in Fig. 4**B**, the surface of UADE or DES-ultrasonic-treated LSP revealed prominent cracks and fissures, which facilitated the release of intracellular metabolites (e.g., flavonoids and polyphenols) by disrupting the integrity of the cell walls. This observation supports the superiority of UADE in inducing structural cell wall disruption, thereby enhancing mass transfer. The ultrasonic treatment significantly improved the tyrosinase inhibition rate, TFC, and TPC of the extracts (Fig. S2). These findings are consistent with established principles of ultrasonic extraction, where cavitation-induced shear forces primarily drive matrix depolymerization [22].

### 3.4 Purification of inhibitors and DES recycling

**Fig. 5:**
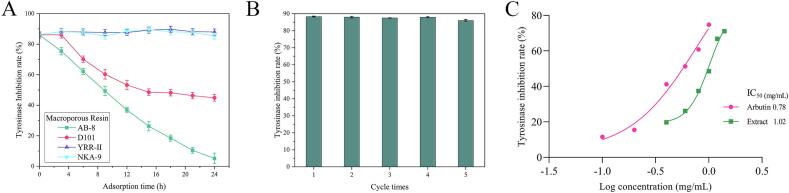
Isolation and purification of tyrosinase inhibitors from LSP extract and recovery of DES. (A) Screening of macroporous resin for inhibitor adsorption, (B) Assessment of DES recyclability, (C) Half-maximal inhibitory concentration (IC_50_) of the purified extract and arbutin. Data are expressed as mean ± SD (n = 3).

Separation and recycling of DESs from extracts present significant challenges for industrial implementation. In this study, macroporous resins were utilized to isolate target compounds and recover DES from the DES-containing extract [47]. As shown in Fig. 5A, the use of AB-8 macroporous resin reduced the inhibition rate to below 5 % within 24 h, demonstrating exceptional adsorption efficiency for the tyrosinase inhibitors. In contrast, D101 resin displayed limited adsorption capacity, with a residual inhibition rate exceeding 40 % after 24 h. Neither YPR-II nor NKA-9 exhibited significant adsorption effects, maintaining inhibition rates between 85 % and 90 % throughout the 24-h period. Consequently, AB-8 resin was selected for further extractions. The aqueous eluate containing DES was concentrated and reused for subsequent extractions. Notably, after five consecutive cycles, no significant changes in DES viscosity were observed, whereas the color shifted from colorless to red, indicating a gradual accumulation of plant pigments in the system. Despite this change, the inhibition rate of the extract remained consistently high (Fig. 5B), confirming the structural integrity of the recycled DES and its potential for sustainable reuse in ultrasound-assisted extraction. The lyophilized extract showed an IC_50_ value of 1.02 mg/mL against tyrosinase (Fig. 5C), which is slightly higher than that of arbutin (IC_50_ = 0.78 mg/mL), suggesting competitive inhibition.

### 3.5 Analysis of LSP extract using UHPLC-QE-Orbitrap-MS

The LSP extract was analyzed using UHPLC-QE-Orbitrap-MS. The total ion chromatograms (TIC) obtained in both positive and negative ion mode are presented in Fig. S1. A total of 185 chemical constituents were identified based on their accurate molecular weights, fragment ion data, and database matching (Tables S12, S13). Flavonoids comprised the predominant chemical class, with 80 species identified, including representative compounds such as procyanidin B1, corymboside, rutin, and luteolin-6-C-glucoside.

### 3.6 Molecular docking and molecular dynamics simulation analysis of potential tyrosinase inhibitors

**Fig. 6:**
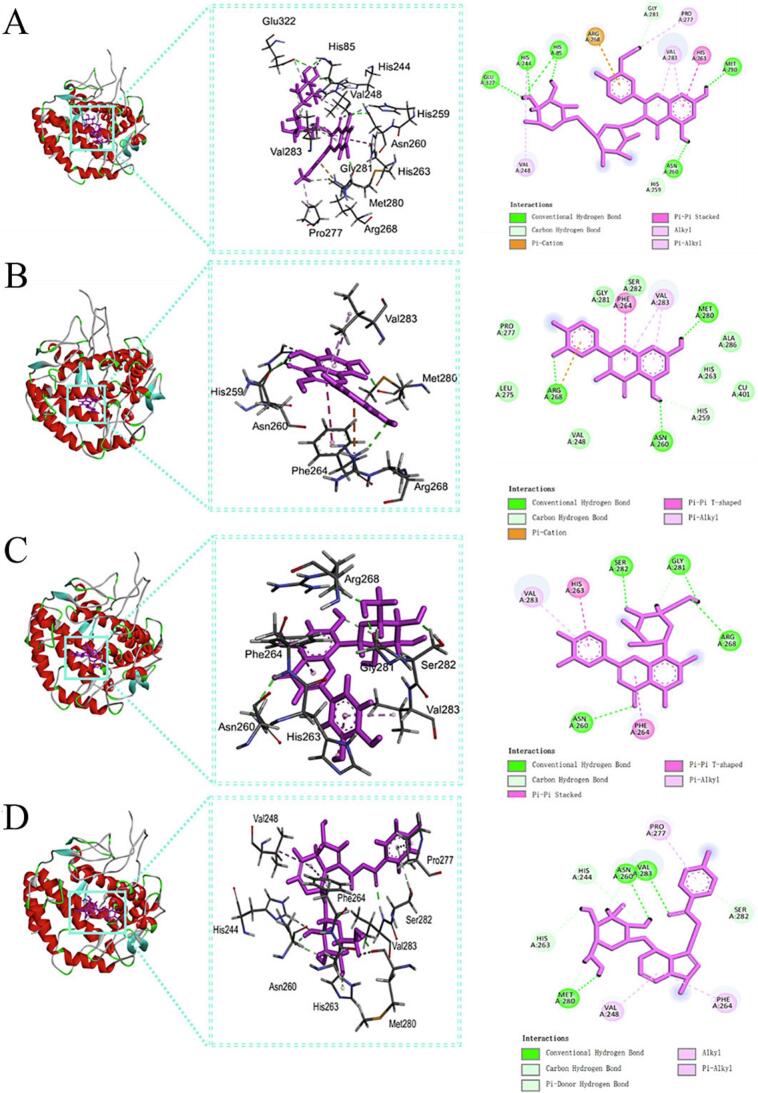
Molecular docking results of the four compounds identified in LSP extract interacting with tyrosinase. Three-dimensional (3D) and two-dimensional (2D) ligand interaction diagrams of tyrosinase with (A) isorhamnetin-3-O-galactoside-6″-rhamnoside, (B) quercetin, (C) luteolin-8-C-glucoside, (D) agnuside.

The top 58 compounds with high relative content identified in the LSP extract (29 in positive ion mode and 29 in negative ion mode) were selected for molecular docking. A comparative analysis revealed an overlap of 2 compounds between the two modes, resulting in a total of 56 unique compounds. Among these, 12 compounds with superior docking scores (＜-5 kcal/mol) were initially selected (Table S14). Subsequent molecular dynamics simulations narrowed the candidate pool to four compounds that formed stable complexes with tyrosinase: isorhamnetin-3-O-galactoside-6″-rhamnoside, quercetin, luteolin-8-C-glucoside, and agnuside. Our analysis also revealed that, although the complexes formed by compounds such as luteolin-7-O-glucoside and glucoluteolin displayed more favorable binding energies, they exhibited significant instability during the molecular dynamics simulations. The molecular docking results indicated that hydrophobic interactions and hydrogen bonds collectively governed the binding process (Fig. 6). All four compounds occupied the enzyme's active site, interacting with surrounding amino acid residues via conventional hydrogen bonds, resulting in the formation of 5 bonds for isorhamnetin-3-O-galactoside-6″-rhamnoside (Fig. 6A), 3 for quercetin (Fig. 6B), 4 for luteolin-8-C-glucoside (Fig. 6C), and 3 for agnuside (Fig. 6D). These hydrogen bonds served as the primary contributors to complex stabilization, while hydrophobic interactions within the enzyme's binding cavity further reinforced the complexes. Notably, isorhamnetin-3-O-galactoside-6″-rhamnoside and quercetin established π-cation interactions with Arg268, enhancing binding stability through electrostatic complementarity.

Molecular dynamics simulations of the tyrosinase-ligand complexes revealed that luteolin-8-C-glucoside and agnuside exhibited superior conformational stability across five key parameters (Fig. S3). The root mean square deviation (RMSD) is a fundamental metric for quantifying the degree of deviation in molecular structures, facilitating the comparison of the molecular conformations of tyrosinase-ligand complexes to the tyrosinase structure. The complexes of tyrosinase with luteolin-8-C-glucoside and agnuside maintained RMSD values below 0.3 Å (Fig. S3**A**), indicating enhanced stability. Additionally, these complexes sustained a high number of persistent hydrogen bonds (Fig. S3**B**). Structural compactness of the tyrosinase-ligand complexes was evidenced by stable radius of gyration (Rg) values (Fig. S3**C**) and reduced solvent-accessible surface area (SASA) values (Fig. S3**D**). A stable Rg value suggests that the protein retains a compact structure throughout the simulation, indicating stable binding conformations of luteolin-8-C-glucoside and agnuside. Concurrently, the decrease in the SASA, which reflects a reduction in the protein's surface area exposed to the solvent, implies that a stable complex forms upon ligand binding, limiting the macromolecule's interactions with other small molecules. In contrast, quercetin displayed larger RMSD fluctuations and the highest SASA value, reflecting increased molecular flexibility that led to unstable binding [48], [49]. The root mean square fluctuation (RMSF) analysis is used for the characterization of structural fluctuations within local regions of tyrosinase after ligand binding. Consistently low RMSF values across all complexes suggested suppressed structural flexibility and high system stability (Fig. S3**E**). Collectively, agnuside and luteolin-8-C-glucoside exhibited optimal binding affinity and conformational stability.

### 3.7 Molecular dynamics analysis of dissolution behavior and intermolecular interaction

**Fig. 7:**
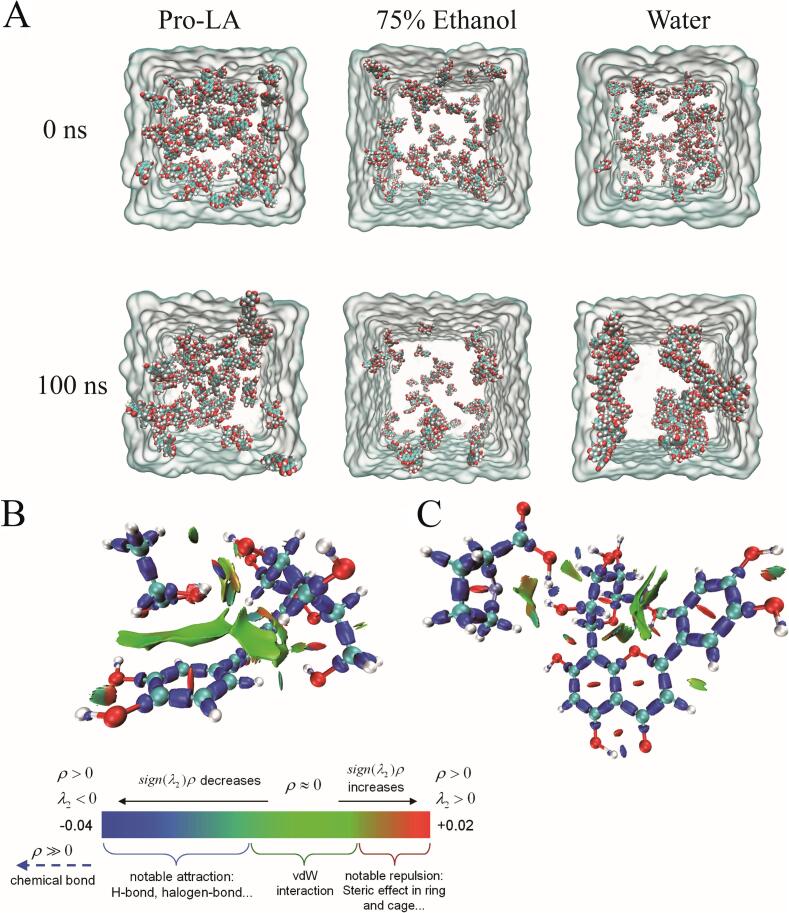
Molecular dynamics analysis of solvation behavior of identified tyrosinase inhibitor and its intermolecular interactions with components of DES. (A) Snapshots of the dissolution process of the identified tyrosinase inhibitors in three distinct solvent systems (DES, 75 % ethanol, and water) from 0 ns to 100 ns, (B) Interaction between lactate and luteolin-8-C-glucoside, (C) Interaction between L-proline and luteolin-8-C-glucoside.

To elucidate the mechanism underlying the efficient extraction of tyrosinase inhibitors by Pro-LA, molecular dynamics (MD) simulations were employed to investigate interactions between four identified inhibitors (isorhamnetin-3-O-galactoside-6″-rhamnoside, quercetin, luteolin-8-C-glucoside, and agnuside) and various solvents (Pro-LA, 75 % ethanol, and water). As shown in Fig. 7A, the spatial distribution of inhibitors within each solvent was monitored over a timeframe of 0–100 ns. The results indicated that tyrosinase inhibitors rapidly clustered in water, while their dispersion was more uniform in both Pro-LA and 75 % ethanol, with optimal uniformity observed specifically in Pro-LA. This disparity in distribution directly correlates with the solvents’ capacity for dissolving the inhibitors. Collectively, the MD simulations confirm that the Pro-LA system enhances solubility and extraction efficiency of tyrosinase inhibitors from LSP by promoting molecular dispersion.

Quantitative studies utilizing density functional theory (DFT) demonstrate that luteolin-8-C-glucoside, a prevalent tyrosinase inhibitor found in LSP, forms extensive weak interactions with LA (Fig. 7B) and Pro (Fig. 7C), which are constituents of DES. These interactions encompass hydrogen bonds, depicted as blue regions in the interaction region index (IRI) analysis, as well as van der Waals forces, represented by green regions. Specifically, the carboxyl and hydroxyl groups of LA form hydrogen bonds with the sugar moiety of luteolin-8-C-glucoside, while its alkyl chain participates in π-alkyl interactions with the benzene ring of the flavonoid aglycone. Simultaneously, Pro contributes to hydrogen bond formation by interacting with the glycoside's oxygen and hydrogen atoms. Furthermore, the pyrrolidine ring of Pro engages in π-π stacking with the aromatic system of the flavonoid. These synergistic effects significantly enhance extraction efficiency, resulting in the exceptional performance of the Pro-LA DES for the selective extraction of tyrosinase inhibitors such as luteolin-8-C-glucoside from LSP.

### 3.8 Whitening efficacy of the extract in zebrafish model

**Fig. 8:**
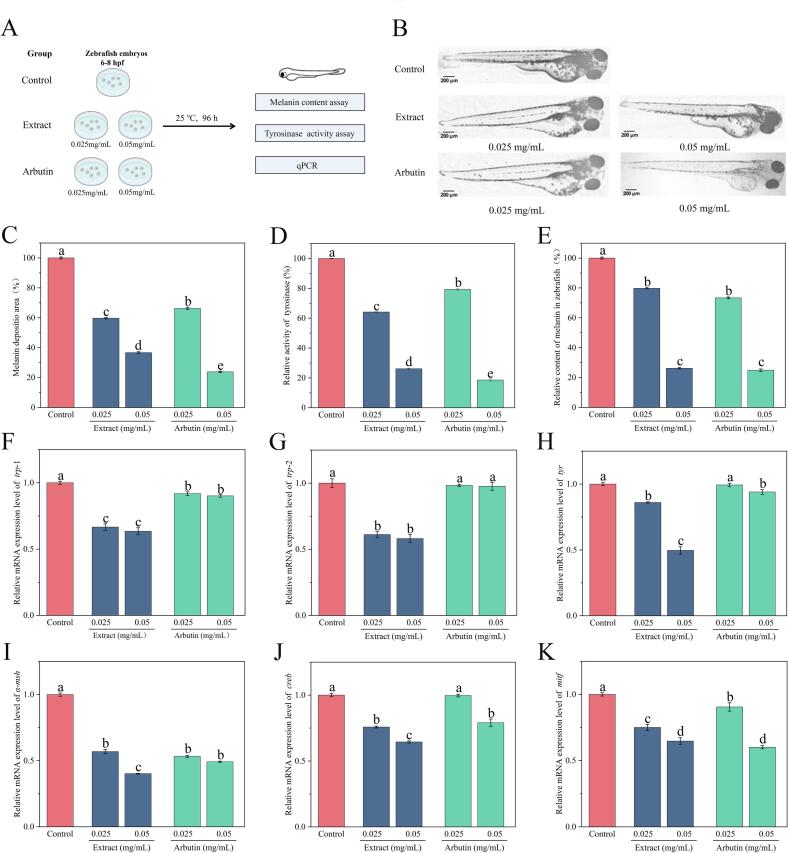
Anti-melanogenic activity of the LSP extract in zebrafish. (A) Schematic diagram of the experimental design, (B) Representative microscopic images of zebrafish larvae (40 × magnification; scale bar: 200 μm), (C) Quantification of melanin deposition area, (D) Relative activity of tyrosinase, (E) Total melanin content, (F-K) Relative mRNA expression levels of melanogenesis-related genes: (F) *trp-1*, (G) *trp-2*, (H) *tyr*, (I) *α-msh*, (J) *creb*, and (K) *mitf*. Data are presented as mean ± SD (n = 3). Different lowercase letters indicate statistically significant differences (*P* < 0.05).

To validate the *in vivo* tyrosinase inhibitory effects of the LSP extract, zebrafish embryos were treated with the extract, using arbutin as a positive control **(**Fig. 8A**)**. As shown in Fig. 8B-C, all treatment groups showed significant reductions in surface melanin area compared to the control group (*P* < 0.05). At a concentration of 0.025 mg/mL, the extract group exhibited a melanin content of 59.71 %, which was lower than that of the arbutin group (66.26 %). At a higher concentration of 0.05 mg/mL, the extract group maintained a melanin content of 37.21 %, exceeding that of arbutin (23.82 %). This divergence underscores a concentration-dependent efficacy divergence between the LSP extract and arbutin. Tyrosinase activity analysis (Fig. 8D) revealed significant enzyme inhibition across all treatment groups compared to the control (*P* < 0.05). At 0.05 mg/mL, the extract-treated group displayed 33.64 % residual tyrosinase activity, which was higher than that of arbutin (26.11 %). Conversely, at 0.025 mg/mL, the relative enzyme activities of the extract and arbutin groups were 64.16 % and 79.73 %, respectively, demonstrating the extract's superior inhibitory efficacy at low concentrations. Analysis of melanin content indicated that at 0.05 mg/mL, the melanin contents of the extract and arbutin groups were 26.20 % and 24.94 % (Fig. 8E), respectively, with no significant difference between the two (*P* > 0.05). Notably, despite higher residual tyrosinase activity, the extract achieved comparable melanin suppression. This observation suggests the potential presence of synergistic anti-melanostatic effects by non-enzymatic pathways, such as antioxidant modulation or inhibition of melanosome transfer.

As illustrated in Fig. 8F-K, the LSP extract significantly downregulated the expression levels of several key genes compared to the control group, including tyrosinase-related protein 1 (*trp-1*) (Fig. 8F), tyrosinase-related protein 2 (*trp-2*) (Fig. 8G), tyrosinase (*tyr*) (Fig. 8H), melanocyte-stimulating hormone (*α-msh*) (Fig. 8I), cAMP response element-binding protein (*creb*) (Fig. 8J), and Microphthalmia-associated transcription factor (MITF) (Fig. 8K). Specifically, the extract significantly suppressed the expression of key genes in the tyrosinase pathway (*trp-1*, *trp-2*, and *tyr*) whereas arbutin exhibited less pronounced effects (Fig. 8F–H). Both the extract and arbutin effectively inhibited the expression of the upstream signaling molecule *α-msh*, with the extract at 0.05 mg/mL demonstrating the most pronounced inhibitory effect (Fig. 8I**).** At 0.025 mg/mL, the extract downregulated *creb* gene similarly to the effects observed with 0.05 mg/mL arbutin (Fig. 8J). For the transcription factor *mitf*, all tested concentrations of extract and arbutin significantly suppressed its expression (Fig. 8K).

## 4 Discussion

Agricultural processing generates substantial quantities of by-products, such as LSP. These by-products are generally rich in bioactive constituents such as polyphenols and alkaloids; however, most have not yet been subjected to high-value utilization [22], [3], [4], [5]. The extraction and isolation of bioactive functional components from such by-products constitute a key pathway for converting agricultural processing residues into valuable resources and increasing their economic value. Conventional aqueous and ethanolic extraction processes typically suffer from low efficiency and high energy consumption [15]. A major bottleneck is that bioactive components such as polyphenols and flavonoids are largely encapsulated within the dense lignocellulosic matrix of by-products such as LSP, which hinders solvent penetration and solute–solvent interactions, thereby limiting further improvements in extraction efficiency [50].

Ultrasound-assisted extraction utilizes cavitation-induced cell wall disruption, mechanical shear forces, and localized heating, making it highly effective for releasing bioactive compounds from LSP (Fig. 1, Fig. 2, Fig. 3, Fig. 4). Furthermore, the acoustic streaming effect of ultrasound can accelerate the diffusion of target components from the solid matrix into the liquid solvent, thereby ensuring that the extraction process remains continuous and efficient. DESs serve as environmentally friendly alternatives that significantly enhance the extraction efficiency of plant constituents compared to traditional solvents [22]. In this study, all DES extracts exhibited higher flavonoid and phenolic contents compared to aqueous and ethanol extracts [51]. SEM images confirmed the disruption of plant cell walls by DES (Fig. 4), which could be attributed to the hydrogen bonding, van der Waals forces, and ionic interactions between DES and cell wall components. This disruption facilitates solvent penetration, significantly enhancing the dissolution of components such as anthocyanins [52], [53], [54]. Overall, the synergistic disruption of plant cell walls by ultrasound and DES is a key factor contributing to the enhanced extraction efficiency [55].

Solvent–solute intermolecular interactions represent another key intrinsic factor influencing extraction efficiency [22], [16], [17], [18], [19]. However, few studies have examined the interactions between tyrosinase-inhibitory components and extraction solvents or assessed how these interactions affect extraction performance [23], [24], [25]. In this study, molecular dynamics simulations showed that the core tyrosinase-inhibitory components tend to spontaneously aggregate into large clusters in conventional solvents, whereas they exhibit substantially higher dispersion stability in the DES system (Fig. 7). This finding elucidates, at the molecular level, the intrinsic mechanism underlying the superior extraction efficiency of the DES system.

Extraction efficiency has been regarded as the primary evaluation metric, whereas extraction selectivity has often been treated as secondary or even neglected. However, at the industrial scale, inadequate selectivity leads to the co-extraction of large amounts of impurities, substantially increasing the cost of downstream separation and purification [56]. Existing research has shown that different DES formulations not only markedly influence the total phenolic and flavonoid contents of extracts but also result in substantial differences in their tyrosinase-inhibitory activity [23], [24], [25]. However, few studies have systematically examined whether DESs can selectively extract target molecules with tyrosinase-inhibitory activity from complex polyphenol and flavonoid mixtures. The results of this study indicate that the Pro–LA DES system enables targeted enrichment of components with high tyrosinase-inhibitory activity. The molecular mechanism of this selective extraction can be explained using computational chemistry methods. Research has shown that DESs and polyphenolic compounds can form hydrogen bonds and electrostatic interactions [57], thereby enhancing solvent–solute interactions and achieving selective enrichment of target compounds [54]. Analysis of intermolecular interactions indicated that hydrogen bonding, van der Waals forces, and π-π stacking are key driving forces in UADE processes examined in this study (Fig. 7). Future studies can employ rational solvent design based on the computational chemistry framework established in this work to develop DES systems with higher extraction efficiency and enhanced targeted enrichment capacity.

Notably, the specific selectivity of DES is not compromised under ultrasound-assisted conditions (Fig. S2). This may be partially attributed to the fact that, under the conditions employed, ultrasound largely preserves the structural integrity of the DES hydrogen-bond network, thereby maintaining its selective solvation ability [54], [55], [56]. However, detailed spectroscopic evidence would be required to confirm this. Overall, the UADE system integrates the dual advantages of ultrasound-enhanced mass transfer and DES-based targeted enrichment. Beyond improving extraction efficiency [55], it enables selective extraction, providing a feasible technical route for the extraction of natural bioactive components [58].

Optimization of extraction process parameters, together with solvent recovery and reuse, is a core strategy for enhancing extraction efficiency, reducing operational costs, and improving the overall economic performance of extraction processes [19], [22]. The molar ratio is a critical factor influencing tyrosinase inhibitor extraction efficiency. Increasing the proportion of carboxylic acids such as lactic acid raises the acidity of DES due to the presence of carboxyl groups. When the ratio of Pro to LA reaches 1:2, the pH of DES drops to 3.1 [59], facilitating the dissolution of lignocellulosic matrices via hydrogen bonding and electrostatic interactions, which enhances solvent penetration. Additionally, the protonation of phenolics may further facilitate their release, thereby improving the extraction of compounds such as proanthocyanidins from the parenchymatous tissue of seed coats [59]. Moreover, excessive acidification can lead to accelerated molecular degradation. Moderate heating can reduce DES viscosity and enhance mass transfer. However, prolonged heating may result in the degradation of thermosensitive compound and the dissolution of impurities [26]. Similarly, extended ultrasonication can promote the dissolution of inhibitors but may also encourage the co-extraction of impurities. Therefore, optimizing processing conditions is essential [26]. Results from solvent recycling experiments demonstrate that the DES system maintains stable extraction performance over successive extraction cycles. Previous research has focused primarily on the effects of solvent reuse on extraction efficiency, whereas changes in extraction selectivity over successive recycling cycles remain poorly characterized [23], [24], [25]. Our results showed that the TPC and TFC levels of the extracts were generally consistent with the trend in tyrosinase inhibition rate (data not shown). This study further suggested that ultrasonic treatment, as a mild physical technique operated under well-controlled temperatures, did not appear to cause catastrophic disruption of the DES hydrogen-bond network, thereby maintaining extraction selectivity [60]. However, the current research is primarily limited to small-scale laboratory trials. Long-term physicochemical stability of DESs under continuous industrial operation, as well as the accumulation of trace impurities during solvent recycling, still require further discussion.

Structural identification of key bioactive molecules is critical for elucidating the material basis of natural product bioactivity and for screening drug lead compounds. Natural tyrosinase inhibitors generally occur as multicomponent mixtures [61], [62], [63]. In this study, UHPLC-QE-Orbitrap-MS analysis, combined with molecular docking and molecular dynamics simulations, was used to systematically characterize the core monomeric constituents responsible for the tyrosinase-inhibitory activity of the extracts. Specifically, quercetin, isorhamnetin-3-O-galactoside-6″-rhamnoside, and luteolin-8-C-glucoside inhibit tyrosinase by directly binding to the enzyme’s active site, a finding supported by both previous reports and the current study [64], [65]. Molecular docking and dynamics simulations further elucidated the binding modes of these small molecules with tyrosinase, analogous to the findings for other plant-derived small molecules [66]. Additionally, we identified one previously unreported tyrosinase inhibitor, agnuside, which was also detected in the ethanolic extract of *Vitex negundo* L. Leaves. Notably, agnuside forms more stable complexes than known inhibitors such as quercetin [67], [68]. Since the activity validation in this study was conducted using a mixture, the inhibitory activity of individual molecules on tyrosinase still requires further experimental validation.

Furthermore, beyond structural identification of the active components, elucidating their targeted enrichment patterns during extraction is essential for understanding the molecular mechanisms that govern extraction performance [69]. However, previous studies on the extraction of tyrosinase inhibitors have focused primarily on interactions between active compounds and their biological target sites, while their interactions with extraction solvents have received comparatively little attention [23], [24], [25]. In this study, computational chemistry approaches were used to analyze the dispersion behavior of the identified active molecules in different solvents and their intermolecular interactions with DES components. The results clarify the intrinsic molecular mechanisms underlying the extraction efficiency and selectivity of the ultrasound-assisted DES system, providing a theoretical basis for the rational optimization of extraction processes.

To further validate the efficacy of the tyrosinase inhibitor extraction method developed in this study, the *in vivo* tyrosinase-inhibitory activity of the obtained extracts was evaluated in zebrafish. Zebrafish embryos serve as an effective vertebrate model for screening melanogenesis inhibitors, allowing for rapid assessment of the effects of compounds on melanin synthesis pathways. Tyrosinase inhibitors may act through diverse mechanisms [9], [10]. RT-qPCR results indicated that a 0.05 mg/mL extract significantly downregulated key melanogenic genes (*trp*-1, *trp*-2, *tyr*), indicating multi-target inhibitory effects through suppression of *α*-*msh* expression and modulation of the *creb* transcriptional activity. In contrast, arbutin did not significantly reduce the gene expression levels of *tyr*, *trp*-1 and *trp*-2. Instead, it reduced melanin synthesis by directly inhibiting tyrosinase activity and decreasing *α*-*msh* levels in melanocytes [70], [71]. This aligns with established evidence that arbutin's depigmentation mechanism involves inhibiting tyrosinase activity rather than suppressing tyrosinase gene expression or protein synthesis [72]. Notably, arbutin downregulated *mitf* expression in our experiments, consistent with a previous report [73]. Moreover, the active components like procyanidin B1 in the LSP powder extract may not only show whitening effects but also regulate the oxidative stress state of skin cells [74]. Procyanidin B1 significantly reduced the levels of phosphorylated p38 MAPK and inhibited the expression of MITF protein [75]. Therefore, the skin-whitening activity of LSP is associated with its multi-target effects, which include the suppression of the *mapk/creb/mitf* axis in melanocytes and inhibition of tyrosinase activity. Collectively, these findings validate the whitening efficacy of tyrosinase inhibitors obtained by UADE from LSP, demonstrating a multi-gene pathway regulation mechanism that aligns with the polypharmacological characteristics of natural compounds [76]. Nevertheless, the confirmation of its specific mechanism of action still requires the use of more complex mouse models to better simulate the intricate physiological environment of human skin.

## 5 Conclusion

Conventional extraction methods typically exhibit low efficiency and poor selectivity, creating a major bottleneck that restricts the high-value utilization of agricultural by-products such as LSP.

To address this challenge, this study developed a UADE system for the targeted isolation of tyrosinase inhibitors from LSP. Following systematic process optimization, the optimal Pro-LA DES system achieved a tyrosinase inhibition rate of 96.51 % and displayed enhanced selectivity. Three known inhibitors (quercetin, isorhamnetin-3-O-galactoside-6′'-rhamnoside, luteolin-8-C-glucoside) and one previously unreported tyrosinase inhibitor, agnuside, were identified in LSP extracts via UHPLC-QE-Orbitrap-MS analysis combined with molecular docking and molecular dynamics simulations. Hydrogen bonding, van der Waals forces, and π-π stacking were identified as the principal intermolecular interactions between inhibitors and DES components, which underpin the dispersion stability of tyrosinase inhibitors in DES and the selective extraction performance of the UADE system. Ultrasonic treatment was verified to markedly disrupt LSP cell wall structure while its mild non-thermal nature preserves the structural integrity of the DES. These findings reveal the underlying mechanisms governing the efficient extraction and targeted enrichment afforded by the UADE system, and provide a robust theoretical basis for the rational design of green extraction processes.

Nevertheless, several limitations remain to be addressed. A rational design strategy for DES systems has yet to be established, and the long-term physicochemical stability of DESs under continuous industrial operation, as well as the accumulation of trace impurities during solvent recycling, has not been evaluated. Future work should proceed along two key directions: (i) developing AI-assisted, rationally designed DES systems with high selectivity, and (ii) constructing pilot-scale continuous processes with optimized industrial ultrasonic parameters and tailored DES regeneration and purification schemes.

Overall, this study develops and validates an efficient and environmentally benign strategy for the valorization of LSP, and offers methodological guidance for the sustainable production of natural tyrosinase inhibitors, with notable scientific value and practical implications for the resource utilization of agricultural by-products.

## Funding sources

This work was supported in part by Postgraduate Scientific Research Innovation Project of Hunan Province, grant number CX20240882; China Postdoctoral Science Foundation (2025 M773733); Postdoctoral Fellowship Program of CPSF (GZC20251959). Open Fund of Yunnan Key Laboratory of Tea Science, 2022YNCX001; Hunan Provincial Natural Science Foundation of China (2026JJ80130); Xiangtan Science and Technology Innovation Project (NC-YB20240024); Top Ten Technical Research Projects in Hunan Province (2024NK1020); Hunan Provincial College Students Innovation Training Program (S144313).

## CRediT authorship contribution statement

**Chengheng Zhang:** Writing – original draft, Software, Formal analysis, Data curation. **Jin Liu:** Writing – review & editing, Visualization, Validation, Data curation. **Jingjing Tan:** Validation, Methodology. **Jiangtao Cai:** Investigation, Data curation. **Ying Long:** Investigation. **Senwen Deng:** Writing – review & editing, Supervision, Project administration, Funding acquisition, Conceptualization. **Shiyin Guo:** Writing – review & editing, Supervision, Project administration. **Changwei Liu:** Writing – review & editing, Resources, Funding acquisition, Conceptualization.

## Declaration of competing interest

The authors declare that they have no known competing financial interests or personal relationships that could have appeared to influence the work reported in this paper.
