# Supplementary material for: Ultrasound-assisted deep eutectic solvent extraction of tyrosinase inhibitors from lotus seed peel powder
Source: Ultrason Sonochem. 2026 Jun 30;131:107944. doi: 10.1016/j.ultsonch.2026.107944 (PMC13355477; doi:10.1016/j.ultsonch.2026.107944)
Supplement: Supplementary Data 1 [file mmc1.docx]

**Appendix A. Supplementary data**

**Ultrasound-assisted deep eutectic solvent extraction of tyrosinase inhibitors from lotus seed peel powder**

Chengheng Zhang ^a†^, Jin Liu ^a†^, Jingjing Tan ^a†^, Jiangtao Cai ^a^, Ying Long ^a^, Senwen Deng ^a,c^*, Shiyin Guo^a^* and Changwei Liu ^a,b^*

^a^ Hunan Engineering Research Center of Lotus Deep Processing and Nutritional Health Sciences, Hunan Key Laboratory of Economic Crops Genetic Improvement and Integrated Utilization, School of Life and Health Sciences, Hunan University of Science and Technology, Xiangtan 411201, China.

^b^ School of Resource & Environment and Safety Engineerng, Hunan University of Science and Technology, Xiangtan 411201, China.

^c^ Yuelushan Laboratory of Hunan Province, Changsha 410004, China.

† These authors contributed equally to this work.

**Corresponding author:**

Senwen Deng ^a,c^*，Shiyin Guo^a^* and Changwei Liu ^a,b^*

1. mail:dswwzls@hnust.edu.cn (S.D.);

[lcvv666@163.com](mailto:lcvv666@163.com) (C.L.);

gsy@hunau.edu.cn(S.G.);

Fig. S1 Total ion chromatograms in positive (A) and negative (B) ion scanning mode.

Fig. S2 The ultrasonic treatment significantly improved the TFC (A), TPC (B), and tyrosinase inhibition rate (C) of the extracts.

Fig. S3 Molecular dynamics simulation of compounds in complex with tyrosinase. (A) RMSD values, (B) HBonds Numbers, (C) Rg values, (D) SASA values, (E) RMSF values.

Table S1 DESs used in this study.

Table S2 Experiment design variables and levels in orthogonal test.

Table S3 Orthogonal experimental design and results.

Table S4 Analysis of variance results of the orthogonal experiment.

Table S5 Experiment design variables and levels of RSM (four factors).

Table S6 Response surface experimental design and results (four factors).

Table S7 Response surface variance analysis results (four factors).

Table S8 Experiment design variables and levels of RSM (three factors).

Table S9 Response surface experimental design and results (three factors).

Table S10 Response surface variance analysis results (three factors).

Table S11 Primers used in this study.

Table S12 The component information of LSP extract in positive ion scanning mode.

Table S13 The component information of LSP extract in negaive ion scanning mode.

Table S14 Molecular docking results and the two-dimensional structure of ligands.


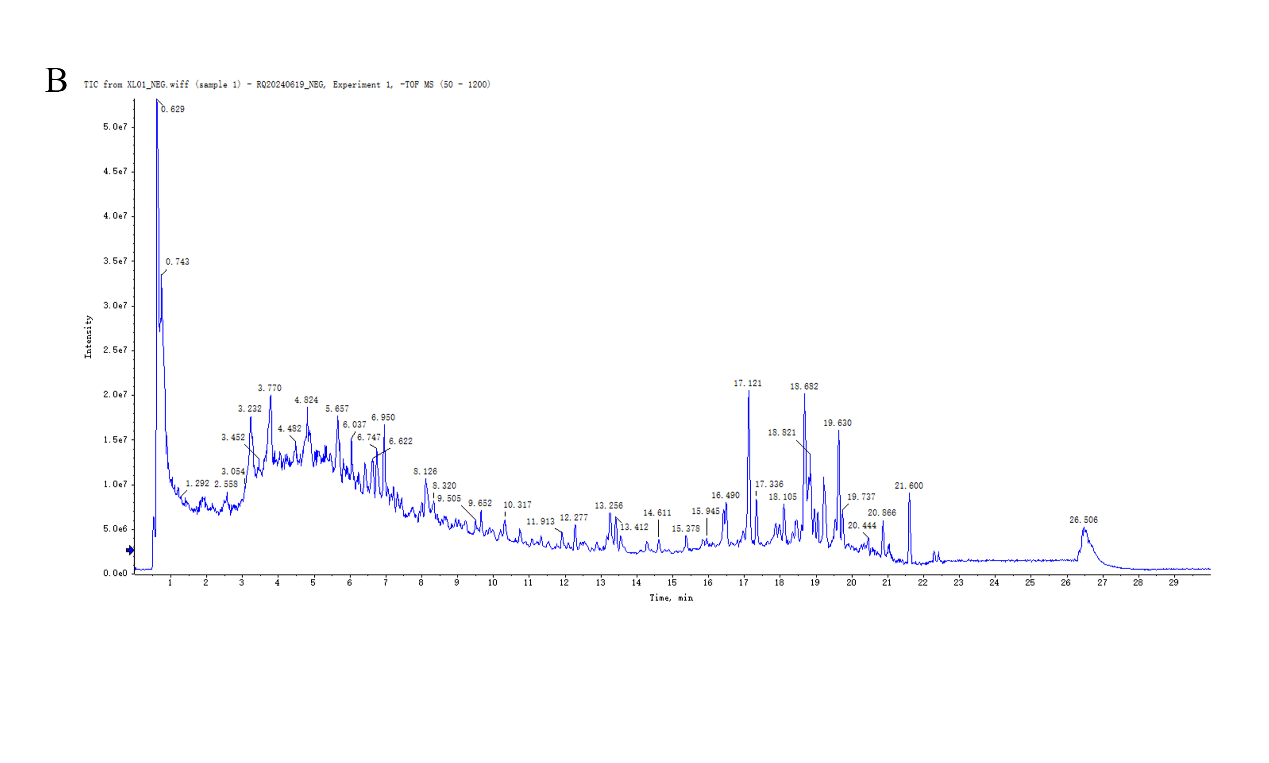

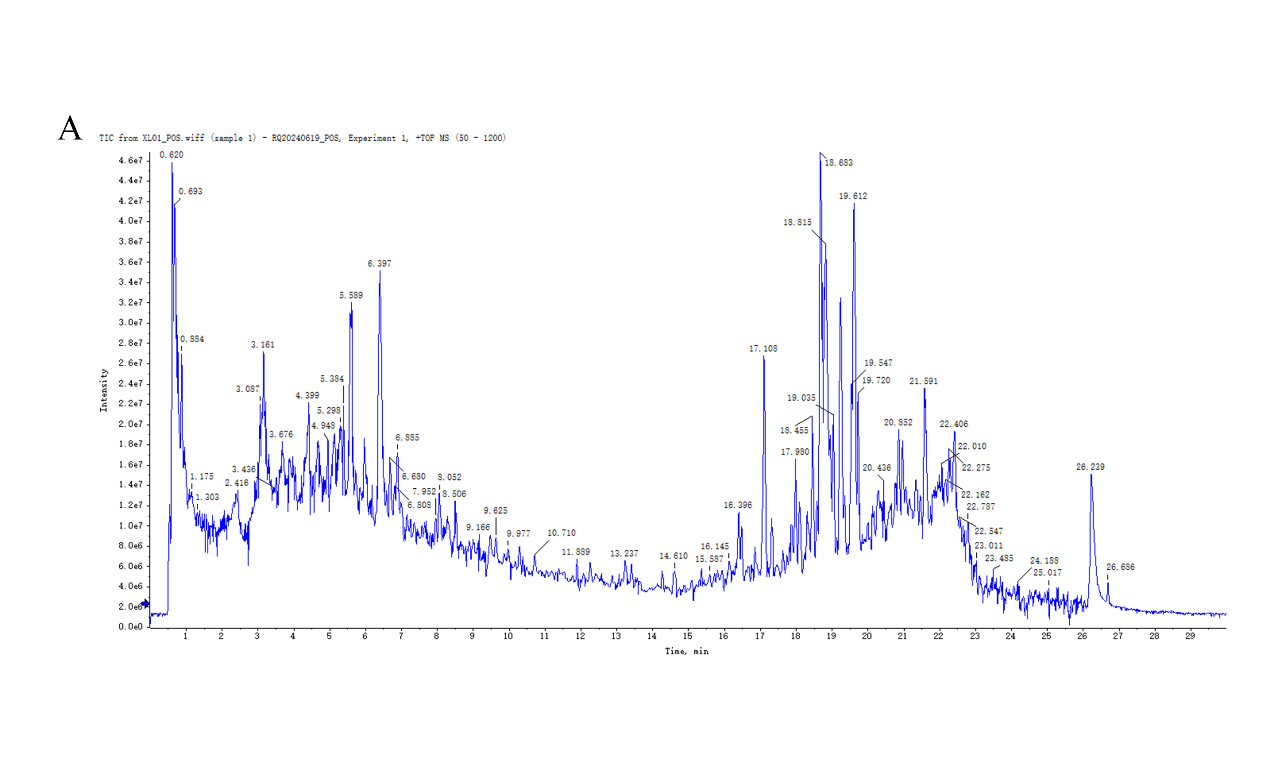
**Fig. S1** Total ion chromatograms in positive (A) and negative (B) ion scanning mode.


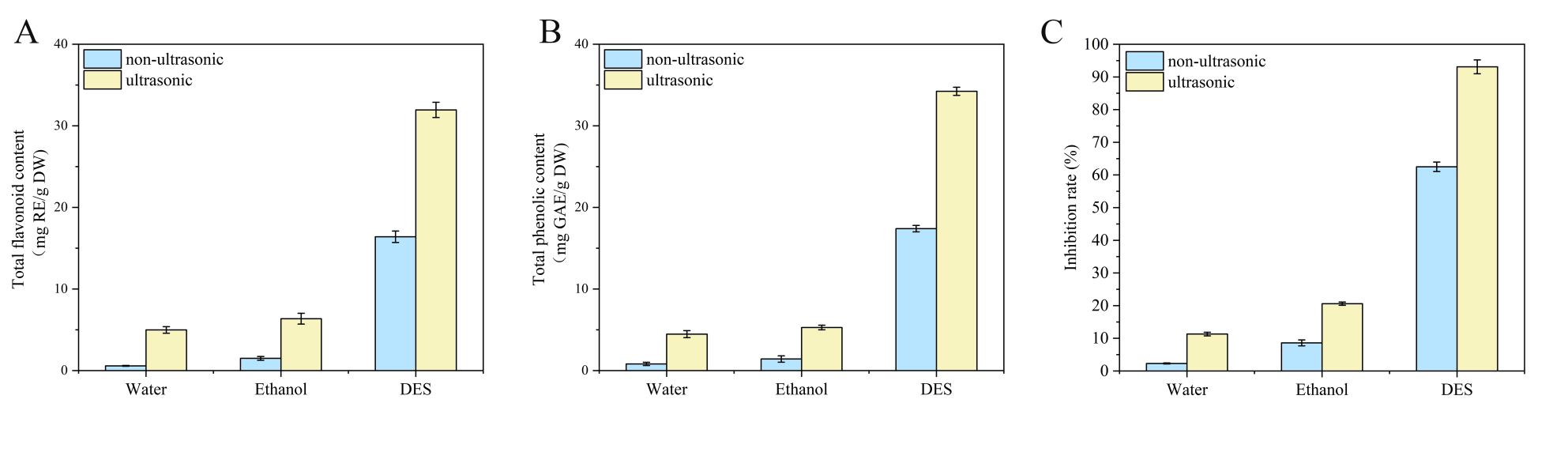
**Fig. S2** The ultrasonic treatment significantly improved the TFC (A), TPC (B), and tyrosinase inhibition rate (C) of the extracts.

**
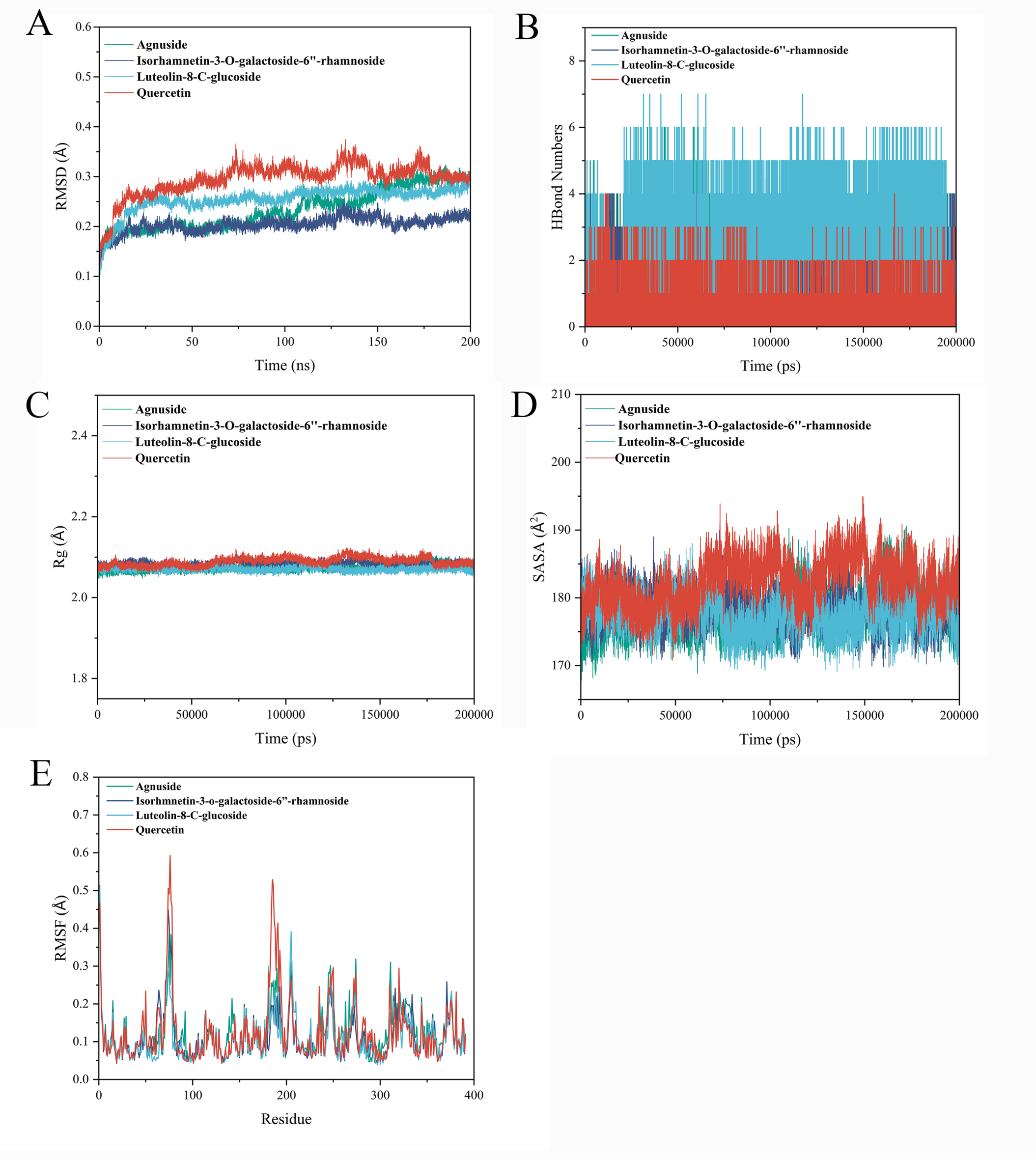
Fig. S3** Molecular dynamics simulation of compounds in complex with tyrosinase. (A) RMSD values, (B) HBonds Numbers, (C) Rg values, (D) SASA values, (E) RMSF values.

**Table S1** DESs used in this study.

| **DESs** | **HBA** | **HBD** | **Molar Ratio** |
| --- | --- | --- | --- |
| ChCl-MA | choline chloride | malic acid | 1：1 |
| ChCl-Glu | choline chloride | glucose | 5：2 |
| ChCl-Xyl | choline chloride | xylitol | 1：1 |
| ChCl-LA | choline chloride | lactic acid | 1：2 |
| ChCl-Urea | choline chloride | urea | 5：2 |
| ChCl-Gly | choline chloride | glycerol | 1：2 |
| ChCl-But | choline chloride | 1,4-butanediol | 1：4 |
| Bet-GLY | betaine | glycerol | 1：1 |
| Bet-CA | betaine | citric acid | 1：1 |
| Bet-LA | betaine | lactic acid | 1：2 |
| Pro-Gly | L-proline | glycerol | 1：2 |
| Pro-Ethy | L-proline | ethylene glycol | 1：2 |
| Pro-LA | L-proline | lactic acid | 1：2 |

**Table S2** Experiment design variables and levels in orthogonal test.

| Factors | Levels | | | |
| --- | --- | --- | --- | --- |
|  | 1 | 2 | 3 | 4 |
| Molar ratio (A) | 1:1 | 1:1.5 | 1:2 | 1:2.5 |
| Water content (B) / % | 10 | 20 | 30 | 40 |
| Ratio of solid to liquid (C) (w/v) | 1:10 | 1:20 | 1:30 | 1:40 |
| Extraction temperature (D) / °C | 40 | 50 | 60 | 70 |
| Ultrasonic power (E) / W | 150 | 200 | 250 | 300 |
| Extraction time (F) /min | 50 | 60 | 70 | 80 |

**Table S3** Orthogonal experimental design and results.

| Test Number | A Molar ratio | B Water content /% | C Solid-liquid ratio (w/v) | D Temperature  /°C | E Power/W | F Time /min | Tyrosinase inhibition/  (%) |
| --- | --- | --- | --- | --- | --- | --- | --- |
| 1 | 3.00 | 1.00 | 2.00 | 3.00 | 1.00 | 4.00 | 80.79 |
| 2 | 4.00 | 2.00 | 1.00 | 3.00 | 4.00 | 4.00 | 88.82 |
| 3 | 2.00 | 4.00 | 1.00 | 3.00 | 1.00 | 2.00 | 74.08 |
| 4 | 1.00 | 2.00 | 2.00 | 2.00 | 2.00 | 2.00 | 73.79 |
| 5 | 4.00 | 3.00 | 1.00 | 2.00 | 2.00 | 4.00 | 81.26 |
| 6 | 4.00 | 4.00 | 2.00 | 1.00 | 1.00 | 3.00 | 88.31 |
| 7 | 2.00 | 3.00 | 2.00 | 4.00 | 2.00 | 1.00 | 86.94 |
| 8 | 4.00 | 2.00 | 4.00 | 3.00 | 3.00 | 1.00 | 87.64 |
| 9 | 2.00 | 1.00 | 4.00 | 2.00 | 4.00 | 3.00 | 91.84 |
| 10 | 3.00 | 2.00 | 4.00 | 4.00 | 1.00 | 2.00 | 92.11 |
| 11 | 2.00 | 3.00 | 3.00 | 4.00 | 1.00 | 4.00 | 89.84 |
| 12 | 2.00 | 2.00 | 3.00 | 1.00 | 3.00 | 4.00 | 84.99 |
| 13 | 3.00 | 4.00 | 2.00 | 2.00 | 3.00 | 4.00 | 84.17 |
| 14 | 1.00 | 4.00 | 1.00 | 4.00 | 3.00 | 1.00 | 74.06 |
| 15 | 4.00 | 1.00 | 3.00 | 4.00 | 4.00 | 2.00 | 87.24 |
| 16 | 1.00 | 2.00 | 3.00 | 2.00 | 1.00 | 3.00 | 71.71 |
| 17 | 3.00 | 3.00 | 1.00 | 1.00 | 4.00 | 3.00 | 91.88 |
| 18 | 3.00 | 2.00 | 1.00 | 4.00 | 2.00 | 3.00 | 91.41 |
| 19 | 1.00 | 4.00 | 4.00 | 4.00 | 4.00 | 4.00 | 58.95 |
| 20 | 1.00 | 1.00 | 4.00 | 1.00 | 2.00 | 4.00 | 66.5 |
| 21 | 1.00 | 1.00 | 1.00 | 1.00 | 1.00 | 1.00 | 59.22 |
| 22 | 2.00 | 2.00 | 2.00 | 1.00 | 4.00 | 1.00 | 89.56 |
| 23 | 3.00 | 4.00 | 3.00 | 2.00 | 4.00 | 1.00 | 86.83 |
| 24 | 1.00 | 3.00 | 3.00 | 3.00 | 3.00 | 3.00 | 64.42 |
| 25 | 4.00 | 3.00 | 4.00 | 2.00 | 1.00 | 1.00 | 93.25 |
| 26 | 4.00 | 1.00 | 2.00 | 4.00 | 3.00 | 3.00 | 82.97 |
| 27 | 4.00 | 4.00 | 3.00 | 1.00 | 2.00 | 2.00 | 78.1 |
| 28 | 3.00 | 3.00 | 4.00 | 1.00 | 3.00 | 2.00 | 88.23 |
| 29 | 1.00 | 3.00 | 2.00 | 3.00 | 4.00 | 2.00 | 69.28 |
| 30 | 2.00 | 1.00 | 1.00 | 2.00 | 3.00 | 2.00 | 85.12 |
| 31 | 3.00 | 1.00 | 3.00 | 3.00 | 2.00 | 1.00 | 85.88 |
| 32 | 2.00 | 4.00 | 4.00 | 3.00 | 2.00 | 3.00 | 86.77 |
| k1 | 67.24 | 79.95 | 80.73 | 80.85 | 81.16 | 82.92 |  |
| k2 | 86.14 | 85 | 81.98 | 83.5 | 81.33 | 80.99 |  |
| k3 | 87.66 | 83.14 | 81.13 | 79.71 | 81.45 | 83.66 |  |
| k4 | 85.95 | 78.91 | 83.16 | 82.94 | 83.05 | 79.42 |  |
| R | 20.42 | 6.1 | 2.43 | 3.79 | 1.89 | 4.25 |  |

**Table S4** Analysis of variance results of the orthogonal experiment.

| Factors | Sum of squares | D/F | F-Value | p-value | Significant |
| --- | --- | --- | --- | --- | --- |
| A | 2259.08 | 3 | 28.77 | < 0.0001 | *** |
| B | 190.74 | 3 | 2.43 | 0.112 |  |
| C | 27.76 | 3 | 0.3535 | 0.7874 |  |
| D | 75.51 | 3 | 0.9617 | 0.4401 |  |
| E | 18.39 | 3 | 0.2342 | 0.8709 |  |
| F | 88.49 | 3 | 1.13 | 0.3743 |  |
| Error | 340.27 | 13 |  |  |  |

*** *P* < 0.001

**Table S5** Variables and levels used in the Response Surface Methodology (RSM) experimental design.

| Levels | Factors | | | |
| --- | --- | --- | --- | --- |
|  | A Molar ratio | B Water content /% | C Extraction temperature /°C | D Extraction time /min |
| -1 | 1.5 | 10 | 40 | 60 |
| 0 | 2 | 20 | 50 | 70 |
| 1 | 2.5 | 30 | 60 | 80 |

**Table S6** Response surface experimental design and results (four factors).

| Test number | A Molar ratio | B Water content /% | C Extraction temperature /°C | D Extraction time /min | Inhibition rate /% |
| --- | --- | --- | --- | --- | --- |
| 1 | 0 | -1 | 0 | -1 | 87.16 |
| 2 | 1 | 0 | 0 | -1 | 85.71 |
| 3 | 1 | 1 | 0 | 0 | 82.31 |
| 4 | 0 | 0 | -1 | -1 | 91.71 |
| 5 | -1 | 0 | 1 | 0 | 72.66 |
| 6 | 1 | 0 | 1 | 0 | 83.16 |
| 7 | 0 | 1 | 1 | 0 | 79.02 |
| 8 | 0 | 0 | 0 | 0 | 93.72 |
| 9 | 0 | -1 | 1 | 0 | 80.83 |
| 10 | 0 | -1 | -1 | 0 | 82.25 |
| 11 | 0 | 0 | 1 | -1 | 79.44 |
| 12 | 0 | 0 | 0 | 0 | 92.88 |
| 13 | -1 | 0 | -1 | 0 | 75.06 |
| 14 | 0 | -1 | 0 | 1 | 93.3 |
| 15 | 0 | 0 | 1 | 1 | 78.78 |
| 16 | 0 | 0 | -1 | 1 | 94.32 |
| 17 | -1 | 0 | 0 | 1 | 71.19 |
| 18 | 1 | 0 | -1 | 0 | 90.41 |
| 19 | 0 | 0 | 0 | 0 | 93.61 |
| 20 | -1 | 1 | 0 | 0 | 67.12 |
| 21 | 1 | 0 | 0 | 1 | 81.55 |
| 22 | 1 | -1 | 0 | 0 | 84.79 |
| 23 | 0 | 1 | 0 | 1 | 79.38 |
| 24 | 0 | 1 | 0 | -1 | 86.51 |
| 25 | 0 | 1 | -1 | 0 | 84.07 |
| 26 | -1 | -1 | 0 | 0 | 78.68 |
| 27 | -1 | 0 | 0 | -1 | 74.03 |

**Table S7** Response surface variance analysis results (four factors).

| Source of variance | Square sum | Degrees of freedom | Mean square | F values | P values | Significance |
| --- | --- | --- | --- | --- | --- | --- |
| Model | 1291.07 | 14 | 92.22 | 6.58 | 0.0012 | ** |
| A Molar ratio | 398.94 | 1 | 398.94 | 28.47 | 0.0002 | *** |
| B Water content | 68.16 | 1 | 68.16 | 4.87 | 0.0476 | * |
| C Extraction temperature | 160.82 | 1 | 160.82 | 11.48 | 0.0054 | ** |
| D Extraction time | 3.04 | 1 | 3.04 | 0.2170 | 0.6497 |  |
| AB | 20.61 | 1 | 20.61 | 1.47 | 0.2485 |  |
| AC | 5.88 | 1 | 5.88 | 0.4197 | 0.5293 |  |
| AD | 0.4356 | 1 | 0.4356 | 0.0311 | 0.8630 |  |
| BC | 3.29 | 1 | 3.29 | 0.2351 | 0.6365 |  |
| BD | 44.02 | 1 | 44.02 | 3.14 | 0.1017 |  |
| CD | 2.67 | 1 | 2.67 | 0.1908 | 0.6700 |  |
| A^2^ | 552.48 | 1 | 552.48 | 39.43 | < 0.0001 | *** |
| B^2^ | 151.75 | 1 | 151.75 | 10.83 | 0.0064 | ** |
| C^2^ | 110.31 | 1 | 110.31 | 7.87 | 0.0159 | * |
| D^2^ | 52.14 | 1 | 52.14 | 3.72 | 0.0777 |  |
| Residual | 168.13 | 12 | 14.01 |  |  |  |
| Lack of fit | 167.71 | 10 | 16.77 | 80.46 | 0.0123 | * |
| Std. Dev. | 3.74 |  | R^2^ | 0.8848 |  |  |
| Mean | 83.10 |  | R^2^Adj | 0.7504 |  |  |
| C.V.% | 4.50 |  | R^2^Pred | 0.3373 |  |  |

* *P* < 0.05; ** *P* < 0.01; ****P* < 0.001.

**Table S8** Experiment design variables and levels of RSM (three factors).

| Levels | Factors | | |
| --- | --- | --- | --- |
|  | A Molar ratio | B Water content /% | C Extraction temperature /°C |
| -1 | 1.5 | 10 | 40 |
| 0 | 2 | 20 | 50 |
| 1 | 2.5 | 30 | 60 |

**Table S9** Response surface experimental design and results (three factors).

| Test  number | A Molar ratio | B Water content /% | C Extraction temperature  /°C | Inhibition rate /% |
| --- | --- | --- | --- | --- |
| 1 | 0 | 0 | 0 | 92.48 |
| 2 | 0 | 0 | 0 | 93.87 |
| 3 | 0 | 0 | 0 | 93.74 |
| 4 | -1 | -1 | 2 | 77.12 |
| 5 | 1 | 0 | -1 | 77.71 |
| 6 | 1 | 0 | -1 | 91.57 |
| 7 | -1 | -1 | 0 | 68.51 |
| 8 | 1 | -1 | 0 | 83.35 |
| 9 | 0 | 0 | 0 | 94.51 |
| 10 | 0 | 1 | 1 | 79.56 |
| 11 | 0 | 0 | 0 | 93.11 |
| 12 | 1 | 1 | 0 | 84.09 |
| 13 | 0 | -1 | 1 | 82.94 |
| 14 | -1 | 0 | 1 | 76.21 |
| 15 | 0 | -1 | -1 | 82.92 |
| 16 | 1 | 0 | 1 | 85.69 |
| 17 | 0 | 1 | -1 | 85.91 |

**Table S10** Response surface variance analysis results (three factors).

| Source of variance | Square sum | Degrees of freedom | Mean square | F values | P values | Significance |
| --- | --- | --- | --- | --- | --- | --- |
| Model | 907.84 | 9 | 100.87 | 71.89 | < 0.0001 | *** |
| A Molar ratio | 254.82 | 1 | 254.82 | 181.59 | < 0.0001 | *** |
| B Water content | 8.53 | 1 | 8.53 | 6.08 | 0.0431 | * |
| C Extraction temperature | 23.50 | 1 | 23.50 | 16.74 | 0.0046 | ** |
| AB | 21.86 | 1 | 21.86 | 15.58 | 0.0056 | ** |
| AC | 4.80 | 1 | 4.80 | 3.42 | 0.1070 |  |
| BC | 10.14 | 1 | 10.14 | 7.23 | 0.0311 | * |
| A^2^ | 246.80 | 1 | 246.80 | 175.88 | < 0.0001 | *** |
| B^2^ | 244.39 | 1 | 244.39 | 174.16 | < 0.0001 | *** |
| C^2^ | 40.23 | 1 | 40.23 | 28.67 | 0.0011 | ** |
| Residual | 9.82 | 7 | 1.40 |  |  |  |
| Lack of fit | 7.42 | 3 | 2.47 | 4.13 | 0.1022 |  |
| Std. Dev. | 1.18 |  | R^2^ | 0.9893 |  |  |
| Mean | 84.9 |  | R^2^Adj | 0.9755 |  |  |
| C.V.% | 1.40 |  | R^2^Pred | 0.8665 |  |  |

* *P* < 0.05; ** *P* < 0.01; ****P* < 0.001.

**Table S11** Primers used in this study.

| Genes | Forward primer | Reverse primer |
| --- | --- | --- |
| *gapdh* | 5’- ACAGCAACACAGAAGACCGT-3’ | 5’- ATACCAGCACCAGCGTCAAA-3’ |
| *α-msh* (*pomca*) | 5’-:GCCCCTGAACAGATAGAGCC-3’ | 5’-CTTGATGGGTCTGCGTTTGC-3’ |
| *creb* | 5’- GCCATGTTCACTTACCATTGAG-3’ | 5’-TTGTAAGAGGCATGTGAGATGG -3’ |
| *mitf* | 5’-TGTACAGCAATCATGCTCTTCC-3’ | 5’-GTCCCCAGCTCCTTAATTCTGTC-3’ |
| *tyr* | 5’-GATCCAGGTCAGCGGTTTGT-3’ | 5’-ACCGATGCGATTATTCCTGCT-3’ |
| *trp1* | 5’-CTCATCATCGTCGCCATC-3’ | 5’-GAACCTCCTGAAGAACACA-3’ |
| *trp2* | 5’-TGGACAGTAAACCCTGGGGA-3’ | 5’-CCGGCAAAGTTTCCAAAGCA-3’ |

**Table S12** The component information of LSP extract in positive ion mode.

| Number | Retention time | Compound | Formula | Area | Relative content (%) | m/z | Classfication |
| --- | --- | --- | --- | --- | --- | --- | --- |
| 1 | 3.0523 | Procyanidin B1 | C_30_H_26_O_12_ | 2.12E+07 | 26.70 | 579.1525 | A |
| 2 | 5.6089 | Corymboside | C_26_H_28_O_14_ | 1.91E+07 | 24.02 | 565.1579 | A |
| 3 | 4.9324 | Vicenin-2 | C_27_H_30_O_15_ | 4410190 | 5.56 | 595.1683 | A |
| 4 | 6.3585 | Vitexin | C_21_H_20_O_10_ | 3105015 | 3.91 | 433.1148 | A |
| 5 | 3.5018 | (2R,3R)-2-(3,4-dihydroxyphenyl)-3,4-dihydro-2H-chromene-3,5,7-triol | C_15_H_14_O_6_ | 3082267 | 3.88 | 291.0877 | A |
| 6 | 5.7213 | Luteolin-8-C-glucoside | C_21_H_20_O_11_ | 2823488 | 3.56 | 449.1101 | A |
| 7 | 18.5861 | Cimiracemoside D | C_37_H_58_O_11_ | 2565061 | 3.23 | 701.376 | F |
| 8 | 6.8948 | Kaempferol-7-O-neohesperidoside | C_27_H_30_O_15_ | 2430285 | 3.06 | 595.1687 | A |
| 9 | 1.7987 | Pantothenic acid | C_9_H_17_NO_5_ | 1372164 | 1.73 | 220.1192 | E |
| 10 | 8.0642 | Tricin 5-glucoside | C_23_H_24_O_12_ | 1153174 | 1.45 | 493.1365 | A |
| 11 | 6.5408 | Quercetin | C_15_H_10_O_7_ | 1017333 | 1.28 | 303.0512 | A |
| 12 | 8.3787 | N-nornuciferine | C_18_H_19_NO_2_ | 901974.9 | 1.14 | 282.1499 | D |
| 13 | 6.5408 | hyperoside | C_21_H_20_O_12_ | 892764.3 | 1.12 | 465.105 | A |
| 14 | 8.0499 | Diosmin | C_28_H_32_O_15_ | 714743.1 | 0.90 | 609.1851 | A |
| 15 | 18.0921 | Kirenol | C_20_H_34_O_4_ | 714221.9 | 0.90 | 339.2523 | F |
| 16 | 23.4565 | Erucamide | C_22_H_43_NO | 611457.4 | 0.77 | 338.3434 | E |
| 17 | 7.3811 | Kaempferol | C_15_H_10_O_6_ | 545226.4 | 0.69 | 287.0558 | A |
| 18 | 4.7906 | Agnuside | C_22_H_26_O_11_ | 4.94E+05 | 0.62 | 467.1559 | F |
| 19 | 4.7624 | Genistein | C_15_H_10_O_5_ | 483168.4 | 0.61 | 271.0621 | A |
| 20 | 7.6523 | Quercetagetin 3,6-dimethyl ether | C_17_H_14_O_8_ | 4.76E+05 | 0.60 | 347.0783 | A |
| 21 | 6.3026 | Violanthin | C_27_H_30_O_14_ | 446901.6 | 0.56 | 579.1736 | A |
| 22 | 18.0921 | Chaulmoogric Acid | C_18_H_32_O_2_ | 438285 | 0.55 | 281.248 | E |
| 23 | 13.0358 | Picfeltarraenin X | C_36_H_54_O_11_ | 413858.8 | 0.52 | 685.3454 | F |
| 24 | 7.6663 | Cyanidin-3-glucoside | C_21_H_21_ClO_11_ | 409133.7 | 0.52 | 449.1104 | A |
| 25 | 7.6238 | Isorhamnetin | C_16_H_12_O_7_ | 4.06E+05 | 0.51 | 317.0675 | A |
| 26 | 6.7248 | Glucoluteolin | C_21_H_20_O_11_ | 394618.5 | 0.50 | 449.1097 | A |
| 27 | 7.7369 | Rhoifolin | C_27_H_30_O_14_ | 374594.3 | 0.47 | 579.1729 | A |
| 28 | 23.1892 | 2-acetoxy-4-pentadecylbenzoic acid | C_24_H_38_O_4_ | 357022.6 | 0.45 | 413.2683 | E |
| 29 | 7.3668 | Homoorientin | C_21_H_20_O_11_ | 352848.1 | 0.44 | 449.1104 | A |
| 30 | 9.1375 | belladonnine | C_34_H_42_N_2_O_4_ | 340009.7 | 0.43 | 543.318 | D |
| 31 | 5.0172 | 4-[4-(beta-D-glucopyranosyloxy)-2-hydroxy-2,6,6-trimethylcyclohexylidene]-3-Buten-2-one | C_19_H_30_O_8_ | 333194.1 | 0.42 | 409.1853 | F |
| 32 | 6.3445 | Rutin | C_27_H_30_O_16_ | 306181.7 | 0.39 | 611.1642 | A |
| 33 | 4.8902 | (-)-Riboflavin | C_17_H_20_N_4_O_6_ | 268708.6 | 0.34 | 377.1479 | G |
| 34 | 19.3559 | echinulin | C_29_H_39_N_3_O_2_ | 221173.5 | 0.28 | 462.3142 | D |
| 35 | 19.9493 | Polyphyllin A | C_27_H_29_F_3_O_6_S | 215787 | 0.27 | 599.3437 | C |
| 36 | 21.8447 | monoolein | C_21_H_40_O_4_ | 212536.8 | 0.27 | 357.3016 | G |
| 37 | 4.9605 | Cimifugin 4'-O-beta-D-glucopyranoside | C_22_H_28_O_11_ | 204453.2 | 0.26 | 469.1704 | B |
| 38 | 9.8562 | 17-Hydroxy-15,16-epoxykauran-18-oic acid | C_20_H_30_O_4_ | 200705.4 | 0.25 | 317.2124 | F |
| 39 | 3.9541 | 3-(4-Hydroxy-3,5-dimethoxyphenyl)-2-propenoic acid | C_11_H_12_O_5_ | 196918.3 | 0.25 | 225.0768 | B |
| 40 | 7.6238 | Isorhamnetin 3-galactoside | C_22_H_22_O_12_ | 185406.5 | 0.23 | 479.1216 | A |
| 41 | 8.0359 | Peonidin-3-O-beta-D-glucoside | C_22_H_21_O_11_ | 172700.4 | 0.22 | 463.1262 | A |
| 42 | 7.3668 | Kaempferol 3-glucuronide | C_21_H_18_O_12_ | 172437.8 | 0.22 | 463.0892 | A |
| 43 | 5.0599 | Myricetin | C_15_H_10_O_8_ | 171135.9 | 0.22 | 319.0466 | A |
| 44 | 19.5380 | Neoruscogenin | C_27_H_40_O_4_ | 170319 | 0.21 | 429.3024 | C |
| 45 | 15.7882 | Cocamidopropyl Betaine | C_19_H_38_N_2_O_3_ | 163791.9 | 0.21 | 343.2976 | D |
| 46 | 3.4734 | trans-Ferulic acid | C_10_H_10_O_4_ | 162566.3 | 0.20 | 177.0543 | B |
| 47 | 7.3811 | Kaempferol-4'-glucoside | C_21_H_20_O_11_ | 156700.6 | 0.20 | 471.0924 | A |
| 48 | 11.6092 | Ophiopogonoside A | C_21_H_38_O_8_ | 150218.1 | 0.19 | 441.2484 | F |
| 49 | 7.3668 | Isorhamnetin-3-O-rutinoside | C_28_H_32_O_16_ | 148388.9 | 0.19 | 647.1638 | A |
| 50 | 6.8663 | Petunidin-3-O-beta-glucopyranoside | C_22_H_23_CO_12_ | 143299.6 | 0.18 | 479.1207 | A |
| 51 | 7.6523 | Syringetin-3-O-glucoside | C_23_H_24_O_13_ | 141161.1 | 0.18 | 509.1314 | A |
| 52 | 12.5459 | Nandrolone | C_18_H_26_O_2_ | 135210.5 | 0.17 | 275.2017 | C |
| 53 | 6.2886 | Naringenin chalcone | C_15_H_12_O_5_ | 134909.7 | 0.17 | 273.0786 | A |
| 54 | 4.4909 | Stepharine | C_18_H_19_NO_3_ | 130084.1 | 0.16 | 298.1446 | D |
| 55 | 4.1093 | Delphinidin-3-O-beta-glucopyranoside | C_21_H_21_ClO_12_ | 129764.9 | 0.16 | 465.106 | A |
| 56 | 7.4096 | Heteratisine | C_22_H_33_NO_5_ | 127465.1 | 0.16 | 392.2453 | D |
| 57 | 9.6233 | Luteolin | C_15_H_10_O_6_ | 126351.3 | 0.16 | 287.0558 | A |
| 58 | 7.3668 | Isorhamnetin-3-O-glucoside | C_22_H_22_O_12_ | 115701.3 | 0.15 | 479.1205 | A |
| 59 | 15.9594 | Piperine | C_17_H_19_NO_3_ | 107650.5 | 0.14 | 286.1452 | D |
| 60 | 11.7697 | Neoechinulin A | C_19_H_21_N_3_O_2_ | 103223.6 | 0.13 | 324.1722 | D |
| 61 | 9.9145 | Grandiflorenic acid | C_20_H_28_O_2_ | 101151.3 | 0.13 | 301.2175 | F |
| 62 | 3.4734 | Kynurenic acid | C_10_H_7_NO_3_ | 100663.4 | 0.13 | 190.0499 | D |
| 63 | 21.6594 | Ceratodictyol | C_19_H_38_O_4_ | 98226.3 | 0.12 | 331.2858 | G |
| 64 | 5.8208 | hydrangeifolin I | C_19_H_28_O_10_ | 95985.38 | 0.12 | 439.1591 | G |
| 65 | 4.4769 | Glycyrrhizin B | C_42_H_62_O_16_ | 95606.55 | 0.12 | 455.119 | F |
| 66 | 1.0461 | Mesalazine | C_7_H_7_NO_3_ | 92201.34 | 0.12 | 154.0501 | E |
| 67 | 14.0708 | Palmitoylcarnitine | C_23_H_45_NO_4_ | 79798.2 | 0.10 | 400.3441 | E |
| 68 | 7.6944 | Naringenin | C_15_H_12_O_5_ | 77423.85 | 0.10 | 273.0763 | A |
| 69 | 3.0523 | Luteolinidin | C_15_H_11_O_5_^+^ | 77341.77 | 0.10 | 271.0602 | A |
| 70 | 5.0599 | Myricetin 3-O-galactoside | C_21_H_20_O_13_ | 75067.47 | 0.09 | 481.1009 | A |
| 71 | 6.1182 | Resveratrol | C_14_H_12_O_3_ | 74389.03 | 0.09 | 229.0876 | G |
| 72 | 5.1450 | Catechin | C_15_H_14_O_6_ | 73655.76 | 0.09 | 291.0871 | A |
| 73 | 3.4734 | Ferulic acid | C_10_H_10_O_4_ | 71237.7 | 0.09 | 195.065 | B |
| 74 | 1.8275 | Trigonelline | C_7_H_7_NO_2_ | 6.95E+04 | 0.09 | 138.0555 | D |
| 75 | 4.1236 | Xanthurenic Acid | C_10_H_7_NO_4_ | 67255.92 | 0.08 | 206.0453 | E |
| 76 | 8.6040 | liriodenine | C_17_H_9_NO_3_ | 59957.57 | 0.08 | 276.067 | D |
| 77 | 6.1610 | Icariside F2 | C_18_H_26_O_10_ | 57489.57 | 0.07 | 403.1594 | G |
| 78 | 6.2886 | Reynosin | C_15_H_20_O_3_ | 57052 | 0.07 | 231.1388 | F |
| 79 | 2.6042 | 3-(4-Hydroxyphenyl-Prop-2-enoic acid | C_9_H_8_O_3_ | 56764.87 | 0.07 | 165.0545 | B |
| 80 | 4.1661 | Sinapine | C_16_H_24_NO_5_ | 55192.15 | 0.07 | 310.1669 | D |
| 81 | 12.2184 | Apigenin | C_15_H_10_O_5_ | 49056.89 | 0.07 | 271.0625 | A |
| 82 | 10.9946 | Sinapinic acid | C_11_H_12_O_5_ | 45621.89 | 0.06 | 225.0759 | B |
| 83 | 6.1752 | Curcumenol | C_15_H_22_O_2_ | 42858.29 | 0.06 | 235.1698 | F |
| 84 | 18.9499 | Oenin | C_23_H_25_O_12_ | 40948.21 | 0.05 | 493.1357 | A |
| 85 | 8.5475 | Isoquinoline | C_9_H_7_N | 38981.43 | 0.05 | 206.1181 | D |
| 86 | 5.3142 | Jervine | C_27_H_39_NO_3_ | 38400.06 | 0.05 | 426.3006 | D |
| 87 | 22.1427 | Nepetin-7-glucoside | C_22_H_22_O_12_ | 37785.04 | 0.05 | 501.104 | A |
| 88 | 7.4519 | Padmatin | C_16_H_14_O_7_ | 36458.84 | 0.05 | 301.0717 | A |
| 89 | 10.8028 | Vanillic acid | C_8_H_8_O_4_ | 36376.23 | 0.05 | 169.049 | E |
| 90 | 11.4214 | 3-Indoleacetic acid | C_10_H_9_NO_2_ | 28454.04 | 0.05 | 176.0711 | E |
| 91 | 7.5239 | Platyphylline | C_18_H_27_NO_5_ | 26584.15 | 0.04 | 338.1963 | D |
| 92 | 6.0332 | Loliolid | C_11_H_16_O_3_ | 23827.15 | 0.03 | 197.1177 | F |
| 93 | 10.8466 | Dihydrosphingosine | C_18_H_39_NO_2_ | 23424.1 | 0.03 | 302.3084 | E |
| 94 | 6.1610 | Deoxycholic acid | C_24_H_40_O_4_ | 20785.02 | 0.03 | 393.2998 | C |
| 95 | 17.3690 | Indole-3-carbinol | C_9_H_9_NO | 19233.42 | 0.03 | 130.0645 | D |
| 96 | 23.2030 | Ergothioneine | C_9_H_15_N_3_O_2_S | 7937.188 | 0.03 | 230.0959 | D |

A: Flavonoids; B: Phenylpropanoid; C: Saponins; D: Alkaloids; E: Organic acids and their derivatives; F: Terpenoids; G: Others

**Table S13** The component information of LSP extract in negaive ion scanning mode.

| Number | Retention time | Compound | Formula | Area | Relative content (%) | m/z | Classfication |
| --- | --- | --- | --- | --- | --- | --- | --- |
| 1 | 3.2172 | Procyanidin B1 | C_30_H_26_O_12_ | 2.12E+07 | 19.82 | 577.1379 | A |
| 2 | 6.4197 | Rutin | C_27_H_30_O_16_ | 1.91E+07 | 7.21 | 609.1493 | A |
| 3 | 6.9540 | Kaempferol-3-O-rutinoside | C_27_H_30_O_15_ | 4410190 | 6.77 | 593.1548 | A |
| 4 | 0.6233 | Raffinose | C_18_H_32_O_16_ | 3105015 | 5.78 | 503.1632 | D |
| 5 | 6.6320 | Quercetin-3-O-galactoside | C_21_H_20_O_12_ | 3082267 | 5.72 | 463.0896 | A |
| 6 | 5.8095 | Luteolin-6-C-glucoside | C_21_H_20_O_11_ | 2823488 | 5.34 | 447.0943 | A |
| 7 | 3.6258 | Epicatechin | C_15_H_14_O_6_ | 2565061 | 4.79 | 289.073 | A |
| 8 | 0.6373 | Sucrose | C_12_H_22_O_11_ | 2430285 | 3.90 | 341.1096 | D |
| 9 | 6.5897 | Quercetin-3-O-glucuronide | C_21_H_18_O_13_ | 1372164 | 3.45 | 477.0691 | A |
| 10 | 20.8655 | y-Linolenic acid | C_18_H_30_O_2_ | 1153174 | 3.06 | 277.2186 | E |
| 11 | 0.8776 | Citric acid | C_6_H_8_O_7_ | 1017333 | 2.70 | 191.0205 | E |
| 12 | 6.4480 | Isovitexin | C_21_H_20_O_10_ | 901974.9 | 2.53 | 431.0993 | A |
| 13 | 5.8947 | Peltatoside | C_26_H_28_O_16_ | 892764.3 | 2.51 | 595.1345 | A |
| 14 | 7.4456 | Kaempferol-3-O-glucoside | C_21_H_20_O_11_ | 714743.1 | 1.93 | 447.0942 | A |
| 15 | 0.6373 | Gluconate | C_6_H_12_O_7_ | 714221.9 | 1.80 | 195.0519 | G |
| 16 | 5.6552 | Myricetin-3-Galactoside | C_21_H_20_O_13_ | 545226.4 | 1.59 | 479.0849 | A |
| 17 | 6.6600 | Cyanidine-3-O-sambubioside | C_26_H_29_O_15_^+^ | 4.94E+05 | 1.35 | 579.1384 | A |
| 18 | 7.4316 | Isorhamnetin-3-O-galactoside-6''-rhamnoside | C_28_H_32_O_16_ | 483168.4 | 1.22 | 623.1656 | A |
| 19 | 8.4329 | Phloretin-2'-O-glucoside | C_21_H_24_O_10_ | 4.76E+05 | 1.18 | 435.1314 | A |
| 20 | 1.6784 | Procyanidin B2 | C_30_H_26_O_12_ | 446901.6 | 1.09 | 577.1378 | A |
| 21 | 7.6866 | Isorhamnetin-3-glucoside | C_22_H_22_O_12_ | 438285 | 0.91 | 477.1061 | A |
| 22 | 6.9962 | Irisxanthone | C_20_H_20_O_11_ | 413858.8 | 0.89 | 435.0941 | G |
| 23 | 6.7855 | Luteolin-7-O-glucoside | C_21_H_20_O_11_ | 409133.7 | 0.87 | 447.0944 | A |
| 24 | 7.7148 | Syringetin-3-O-glucoside | C_23_H_24_O_13_ | 4.06E+05 | 0.82 | 507.1169 | A |
| 25 | 22.4182 | Trans-Vaccenic acid | C_18_H_34_O_2_ | 394618.5 | 0.80 | 281.2499 | E |
| 26 | 18.8746 | Maslinic acid | C_30_H_48_O_4_ | 374594.3 | 0.74 | 471.3497 | E |
| 27 | 5.6692 | Isoquercetrin | C_21_H_20_O_12_ | 352848.1 | 0.73 | 463.0904 | A |
| 28 | 1.7072 | Gallocatechin | C_15_H_14_O_7_ | 340009.7 | 0.67 | 305.0679 | A |
| 29 | 9.6385 | Quercetin | C_15_H_10_O_7_ | 333194.1 | 0.54 | 301.0363 | A |
| 30 | 1.9037 | Pantothenic acid | C_9_H_17_NO_5_ | 306181.7 | 0.52 | 218.104 | E |
| 31 | 8.0958 | Diosmetin-7-O-neohesperidoside | C_28_H_32_O_15_ | 268708.6 | 0.51 | 607.1712 | A |
| 32 | 1.6076 | 3,4-Dihydroxybenzoic acid | C_7_H_6_O_4_ | 221173.5 | 0.48 | 153.0197 | C |
| 33 | 7.6300 | Dihydrokaempferol | C_15_H_12_O_6_ | 204453.2 | 0.45 | 287.0563 | A |
| 34 | 4.7073 | Catechin | C_15_H_14_O_6_ | 200705.4 | 0.44 | 289.073 | A |
| 35 | 7.4316 | Kaempferol-3-O-glucuronoside | C_21_H_18_O_12_ | 196918.3 | 0.42 | 461.0739 | A |
| 36 | 0.7929 | Adenosine-3-monophosphate | C_10_H_14_N_5_O_7_P | 185406.5 | 0.39 | 346.0562 | G |
| 37 | 6.3490 | Taxifolin | C_15_H_12_O_7_ | 172700.4 | 0.34 | 303.0514 | A |
| 38 | 11.2628 | Kaempferol | C_15_H_10_O_6_ | 172437.8 | 0.32 | 285.0414 | A |
| 39 | 3.0770 | Protocatechuic aldehyde | C_7_H_6_O_3_ | 171135.9 | 0.31 | 137.0247 | C |
| 40 | 7.7148 | Luteolin-4'-O-glucoside | C_21_H_20_O_11_ | 170319 | 0.29 | 447.0952 | A |
| 41 | 2.0304 | 2,5-Dihydroxybenzoic acid | C_7_H_6_O_4_ | 163791.9 | 0.23 | 153.0198 | C |
| 42 | 9.6667 | Luteolin | C_15_H_10_O_6_ | 162566.3 | 0.22 | 285.0413 | A |
| 43 | 8.1936 | Bracteatin | C_15_H_10_O_7_ | 156700.6 | 0.21 | 301.0351 | C |
| 44 | 3.6116 | Dihydrocoumaroyl Hexoside | C_15_H_20_O_8_ | 150218.1 | 0.19 | 327.1095 | A |
| 45 | 7.8274 | Myricetin | C_15_H_10_O_8_ | 148388.9 | 0.18 | 317.0302 | A |
| 46 | 5.0889 | Taxifolin-3-glucoside | C_21_H_22_O_12_ | 143299.6 | 0.17 | 465.1052 | A |
| 47 | 11.4226 | 4',5,7-trihydroxy-3,6-dimethoxyflavone | C_17_H_14_O_7_ | 141161.1 | 0.17 | 329.0679 | A |
| 48 | 5.1739 | Myricetin-3-O-galactoside | C_21_H_20_O_13_ | 135210.5 | 0.16 | 479.0842 | A |
| 49 | 5.5991 | 5-Methoxysalicylic acid | C_8_H_8_O_4_ | 134909.7 | 0.15 | 167.0354 | C |
| 50 | 4.7641 | Coumaroyl quinic acid | C_16_H_18_O_8_ | 130084.1 | 0.14 | 337.0944 | E |
| 51 | 3.6970 | 2-Isopropylmalic acid | C_7_H_12_O_5_ | 129764.9 | 0.14 | 175.0621 | E |
| 52 | 18.5691 | Corosolic acid | C_30_H_48_O_4_ | 127465.1 | 0.14 | 471.3495 | F |
| 53 | 7.7288 | Spiraeoside | C_21_H_20_O_12_ | 126351.3 | 0.14 | 463.0902 | A |
| 54 | 2.7954 | 3-Hydroxycinnamic acid | C_9_H_8_O_3_ | 115701.3 | 0.14 | 163.0406 | C |
| 55 | 6.9400 | Petunidin-3-O-beta-glucopyranoside | C_22_H_23_ClO_12_ | 107650.5 | 0.13 | 477.1056 | A |
| 56 | 2.0304 | Catechol | C_6_H_6_O_2_ | 101151.3 | 0.13 | 109.0296 | C |
| 57 | 21.3266 | 9-Trans-Palmitelaidic acid | C_16_H_30_O_2_ | 100663.4 | 0.13 | 253.2186 | E |
| 58 | 21.1018 | Myristic acid | C_14_H_28_O_2_ | 95985.38 | 0.13 | 227.2029 | E |
| 59 | 6.2064 | Piceid | C_20_H_22_O_8_ | 95606.55 | 0.12 | 389.1253 | C |
| 60 | 3.6116 | Methyl gallate | C_8_H_8_O_5_ | 92201.34 | 0.11 | 183.0309 | C |
| 61 | 11.6523 | Isorhamnetin | C_16_H_12_O_7_ | 79798.2 | 0.11 | 315.0519 | A |
| 62 | 0.7080 | Fumaric acid | C_4_H_4_O_4_ | 77423.85 | 0.11 | 115.0039 | E |
| 63 | 23.2597 | Methyl Heptadecanoic acid | C_18_H_36_O_2_ | 77341.77 | 0.10 | 283.2653 | E |
| 64 | 6.2492 | Sinapic acid | C_11_H_12_O_5_ | 75067.47 | 0.10 | 223.0619 | B |
| 65 | 8.4329 | Phloretin | C_15_H_14_O_5_ | 73655.76 | 0.09 | 273.0778 | A |
| 66 | 19.6849 | Linoleic acid | C_18_H_32_O_2_ | 71237.7 | 0.08 | 279.2335 | E |
| 67 | 9.5257 | Abscisic acid | C_15_H_20_O_4_ | 6.95E+04 | 0.08 | 263.1297 | E |
| 68 | 0.8776 | Mesaconic acid | C_5_H_6_O_4_ | 67255.92 | 0.08 | 129.0195 | E |
| 69 | 1.0946 | Gallic acid | C_7_H_6_O_5_ | 59957.57 | 0.07 | 169.0148 | C |
| 70 | 9.1550 | Feruloyltyramine | C_18_H_19_NO_4_ | 57489.57 | 0.07 | 312.1252 | C |
| 71 | 1.4331 | Cinnamic acid | C_9_H_8_O_2_ | 57052 | 0.07 | 147.0457 | E |
| 72 | 1.1088 | Pyrogallol | C_6_H_6_O_3_ | 56764.87 | 0.06 | 125.0244 | C |
| 73 | 3.5695 | Isoferulic acid | C_10_H_10_O_4_ | 55192.15 | 0.06 | 193.0511 | B |
| 74 | 19.5307 | Trehalose | C_12_H_22_O_11_ | 49056.89 | 0.04 | 341.1108 | D |
| 75 | 10.7908 | Endocrocin | C_16_H_10_O_7_ | 45621.89 | 0.04 | 313.0363 | G |
| 76 | 6.2064 | trans-Resveratrol | C_14_H_12_O_3_ | 42858.29 | 0.04 | 227.072 | C |
| 77 | 4.9479 | 2-Hydroxy-4-methylpentanoic acid | C_6_H_12_O_3_ | 40948.21 | 0.03 | 131.072 | E |
| 78 | 10.3753 | Aleuretic Acid | C_16_H_32_O_5_ | 38981.43 | 0.03 | 303.2188 | E |
| 79 | 11.4516 | Isokaempferide | C_16_H_12_O_6_ | 37785.04 | 0.03 | 299.0575 | A |
| 80 | 11.0346 | Apigenin | C_15_H_10_O_5_ | 37590.05 | 0.03 | 269.0468 | A |
| 81 | 3.3717 | γ-Glutamylleucine | C_11_H_20_N_2_O_5_ | 36458.84 | 0.03 | 259.1292 | E |
| 82 | 24.3600 | Arachidic acid | C_20_H_40_O_2_ | 36376.23 | 0.03 | 311.2959 | E |
| 83 | 17.2312 | Mitragynine | C_23_H_30_N_2_O_4_ | 28454.04 | 0.02 | 443.221 | G |
| 84 | 20.7387 | Palmitic Acid | C_16_H_32_O_2_ | 27628.65 | 0.02 | 255.2337 | E |
| 85 | 4.9479 | 6,7-Dihydroxycoumarin | C_9_H_6_O_4_ | 26584.15 | 0.02 | 177.0207 | B |
| 86 | 25.8644 | Behenic acid | C_22_H_44_O_2_ | 23827.15 | 0.02 | 339.3281 | E |
| 87 | 2.2430 | 3-Hydroxybenzaldehyde | C_7_H_6_O_2_ | 23424.1 | 0.02 | 121.0296 | C |
| 88 | 7.0941 | 9-(2,3-dihydroxypropoxy)-9-oxononanoic acid | C_12_H_22_O_6_ | 19233.42 | 0.02 | 261.1349 | E |
| 89 | 6.7713 | Indole-3-carboxylic acid | C_9_H_7_NO_2_ | 7937.188 | 0.02 | 160.0404 | E |

A: Flavonoids; B: Phenylpropanoid; C: Non-flavonolic phenols; D: Saccharide; E: Organic acids and their derivatives; F: Terpenoids; G: Others

**Table S14** Molecular docking results and the two-dimensional structure of ligands.

| Compounds | PubChem CID | Docking score（kcal/mol） | 2D structure |
| --- | --- | --- | --- |
| Procyanidin-B2 | 122738 | -5.82 |  |
| isorhamnetin-3-O-galactoside-6 rhamnoside | 6223069 | -8.02 |  |
| Quercetin | 280343 | -6.711 |  |
| Gallocatechin | 65084 | -6.187 |  |
| Luteolin-7-O-glucoside | 280637 | -8.528 |  |
| Rutin | 5280805 | -8.032 |  |
| Agnuside | 442416 | -6.286 |  |
| Chaulmoogric Acid | 441446 | -5.313 |  |
| Glucoluteolin | 5280637 | -8.528 |  |
| Kirenol | 15736732 | -5.322 |  |
| Luteolin-8-C-glucoside | 5281675 | -8.462 |  |
| Vitexin | 5280441 | -7.622 |  |
